# A novel method for comparison of arterial remodeling in hypertension: Quantification of arterial trees and recognition of remodeling patterns on histological sections

**DOI:** 10.1371/journal.pone.0216734

**Published:** 2019-05-21

**Authors:** Alex A. Gutsol, Paula Blanco, Svetlana I. Samokhina, Sergey A. Afanasiev, Chris R. J. Kennedy, Sergey V. Popov, Kevin D. Burns

**Affiliations:** 1 Kidney Research Centre, Ottawa Hospital Research Institute, ON, Canada; 2 Department of Pathology and Laboratory Medicine, University of Ottawa, ON, Canada; 3 Tomsk State University, Tomsk, Russian Federation; 4 Cardiology Research Institute, Tomsk, Russian Federation; 5 Division of Nephrology, Department of Medicine, University of Ottawa, ON, Canada; Temple University School of Medicine, UNITED STATES

## Abstract

Remodeling of spatially heterogeneous arterial trees is routinely quantified on tissue sections by averaging linear dimensions, with lack of comparison between different organs and models. The impact of experimental models or hypertension treatment modalities on organ-specific vascular remodeling remains undefined. A wide variety of arterial remodeling types has been demonstrated for hypertensive models, which include differences across organs. The purpose of this study was to reassess methods for measurement of arterial remodeling and to establish a morphometric algorithm for standard and comparable quantification of vascular remodeling in hypertension in different vascular beds. We performed a novel and comprehensive morphometric analysis of terminal arteries in the brain, heart, lung, liver, kidney, spleen, stomach, intestine, skin, skeletal muscle, and adrenal glands of control and Goldblatt hypertensive rats on routinely processed tissue sections. Mean dimensions were highly variable but grouping them into sequential 5 μm intervals permitted creation of reliable linear regression equations and complex profiles. Averaged arterial dimensions demonstrated seven remodeling patterns that were distinct from conventional inward-outward and hypertrophic-eutrophic definitions. Numerical modeling predicted at least nineteen variants of arterial spatial conformations. Recognition of remodeling variants was not possible using averaged dimensions, their ratios, or the remodeling and growth indices. To distinguish remodeling patterns, a three-dimensional modeling was established and tested. The proposed algorithm permits quantitative analysis of arterial remodeling in different organs and may be applicable for comparative studies between animal hypertensive models and human hypertension. Arterial wall tapering is the most important factor to consider in arterial morphometry, while perfusion fixation with vessel relaxation is not necessary. Terminal arteries in organs undergo the same remodeling pattern in Goldblatt rats, except for organs with hemodynamics affected by the arterial clip. The existing remodeling nomenclature should be replaced by a numerical classification applicable to any type of arterial remodeling.

## Introduction

The importance of understanding the pathogenesis of hypertension is undisputed, and despite recent decreases in mortality due to heart disease and stroke, the burden of disease remains high. Based on data from 2018, 33% of adults in North America have hypertension, but only 53% of those with documented hypertension have their condition controlled to target levels[1]. An important feature of hypertensive angiopathy is vascular remodeling: a complex structural and spatial modification in small arteries that is of crucial functional consequence since it alters peripheral resistance and impairs contractility[2],[3],[4],[5].

The features of arterial remodeling in hypertension have been extensively studied[3],[4],[6],[7]. To date however, there are no data demonstrating how arterial remodeling in spontaneously hypertensive rats is distinct from hypertension due to angiotensin II or deoxycorticosterone acetate infusion, inhibition of nitric oxide synthesis, or Goldblatt’s model. The utility of animal models must be based on their resemblance to human pathology, yet commonly used measures are unable to quantify how arterial remodeling in experimental animals corresponds to arterial remodeling in humans. Thus, the well-established clinical concept of target organ damage in hypertension[8] has yet to be supported by data indicating that arterial remodeling is more extensive in the kidney or heart, for example, compared to the skin, liver, or other organs.

In the majority of studies complex three-dimensional (3D) intra-organ arterial trees have been quantified by simple measures first described more than 80 years ago[9], consisting of the wall-to-lumen ratio (WLR), and/or the mean values for a variety of arterial dimensions, e.g., external diameter (ED), internal diameter (ID), wall thickness (WTh), external perimeter (EP), internal perimeter (IP), media cross sectional area (MCSA), lumen cross sectional area (LCSA), total cross sectional area and internal radius. These measures are highly variable and inconsistent, and do not permit comparison of remodeling between different organs, hypertension models or species. Presumably, that quantification of tapered 3D intra-organ arterial trees on tissue sections is oversimplified, and conventional averaging of dimensions in vessel morphometry produce incomparable stochastic results. Modern micro-computed tomography achieves 3D images of peripheral vessels with high resolution: ~ 2.5 μm voxel size. However, in this technique, detailed microstructure cannot be described since only the contrast-filled lumen is visualised.

Arterial wall remodeling patterns have been classified as hypertrophic, hypotrophic or eutrophic, associated with inward narrowing or outward widening of the lumen[6],[10]. To date, however, there is significant discrepancy with regards to the type of remodeling that develops in terminal arteries (TAs) of different organs in various hypertensive models. In spontaneously hypertensive rats for example, renal arterioles may demonstrate remodeling characterized as outward hypertrophic[11],[12],[13], inward hypertrophic[14], hypertrophic[12] or no change[13],[15]. Some authors conclude that renal arteries > 60 μm do not develop remodeling[16], while others suggest an absence of remodeling for smaller arteries < 60 μm[15]. Mesenteric arteries may show inward hypertrophy or no change[11],[17]. Similar apparent contradictions appear in other hypertensive models[18],[19],[20].

Such discrepancies could arise since arterial remodeling has previously been classified using empirical drawings, without precise quantitative analysis[10]. Since it was introduced twenty two years ago, the classification has been extensively reviewed[5],[21],[22], but has not been challenged with quantitative methods. Furthermore, frequently used parameters, such as WLR, remodeling index (RI), and growth index (GI) have not been rigorously tested as markers of remodeling. We therefore set out to i) study the classification of arterial remodeling patterns in hypertension, using mathematical methods, and ii) elucidate how arterial remodeling differs across a variety of organs. An algorithm for arterial remodeling assessment was developed, and we then determined if it could distinguish variants of remodeling in different organs within one model of hypertension in Goldblatt one-kidney one-clip (1K1C) rats. We hypothesized that, if conventional averaging was avoided, all organs would show the same arterial remodeling pattern. We also hypothesized distinct remodeling patterns for the adrenal gland and kidney, where the 1K1C model creates particular hemodynamic conditions. In this model, arteries within the adrenal glands experience the effects of an activated renin-angiotensin system (RAS), similar to other organs, but also experience enhanced flow due to diversion of blood from the main renal artery as a result of distal stenosis or ligation, similar to hemodynamic models of overflow[23],[24]. The remaining kidney also experiences the vasoconstrictive effects of an activated RAS, but under low blood flow due to the clipped renal artery[25],[26], that corresponds to low blood flow models[23],[27].

We also determined if examination of random tissue sections can provide useful information in studying hypertension, compared to use of arterial myography[3],[28]. In this regard, the vast majority of data from *in vitro* myography experiments has been generated from dissected mesenteric arteries, and may not be applicable to other organs. Finally, studies have recommended that arterial morphometry should only be performed on perfusion-fixed organs with pharmacologically relaxed vessels[28],[29]. We therefore examined if routine immersion fixation is appropriate for arterial remodeling analysis to elucidate comparisons between animal and human samples, since for the latter immediate relaxation and perfusion fixation are not practical in general.

## Material and methods

Experimental protocols were approved by the Animal Ethics Committee at the University of Ottawa and performed according to the recommendations of the Canadian Council for Animal Care. Analyses in normal rats were performed on 20 male Wistar rats (Charles River Laboratories, Montreal, Québec, Canada), age 20–25 months, and weighing 600–800 g. Five normal male C57BL6 mice (age 20–30 weeks) were used for comparative morphometry. Goldblatt 1K1C hypertension was induced in 20 male Wistar rats (Charles River Laboratories), age 20–25 months, weighing 600–800 gm. Under isoflurane anesthesia a silver clip with internal diameter 0.26 mm was placed around the left renal artery and the right kidney was removed. On both sides the manipulation was distal to the adrenal arteries to keep them intact. The control sham-operated group (5 animals) was kept under the same living conditions as the experimental animals. Systolic blood pressure was measured weekly by tail-cuff plethysmography. After 60 days rats were euthanized with an intraperitoneal overdose of sodium pentobarbital. The brain, lung, heart, stomach, liver, small intestine, spleen, kidneys, adrenals, hip skeletal muscle, and tail skin were excised and immersion-fixed in 10% buffered formalin for 24 h, dehydrated and embedded in paraffin. Tissue blocks were specifically oriented according to the known anatomical distribution of arteries in order to obtain predominantly cross-circular arterial sections. Histological sections (5 μm thickness) were stained with hematoxylin-eosin and Masson’s trichrome. In sections of three to four blocks from each organ, small arteries and arterioles were traced by visual scanning of the entire section. Only vessels with a long- to short-axis ratio < 1.50 were measured. In this way, the error associated with calculating the diameter by averaging the maximum and minimum diameters was minimized (<3%)[30]. The ED and ID were measured in arteries if the profile had a visibly non-interrupted circular or ellipsoid shape. From those two values the derivatives were calculated as follows: WTh = (ED–ID)/2; WLR = WTh /ID; media cross sectional area (MCSA) = π* (ED^2^/2-ID^2^/2). Microscopy was performed with a Zeiss AX10 microscope (Oberkochen, Germany) and images were analyzed by ImagePro Plus software (Media Cybernetics, Bethesda, MD, USA).

Descriptive statistics calculated mean, standard deviation, standard error of mean, coefficient of variation, and frequency distribution to build histograms and complex profiles. Distribution was assessed by Kolmogorov-Smirnov, D’Agostino & Pearson and Shapiro-Wilk tests. Outliers were identified by the combined robust regression and outlier removal method. Acquired linear regression equations were tested to determine if slopes and intercepts were significantly different (P < 0.05) and the goodness of fit coefficient *r*^*2*^ was counted for each equation. Nonlinear regression was approximated with exponential growth equations and the goodness of fit coefficient *R*^*2*^ was counted for each equation. Extra sum-of-squares F-test and Akaike’s Information Criteria were used to compare the best-fit values between nonlinear regression equations (P < 0.05). Statistical analyses were performed with GraphPad Prism software (GraphPad Software, La Jolla, CA, USA). Modeling procedures were made with MathCAD version 15.0 (Parametric Technology Corporation, Needham, MS, USA). Three-dimensional graphs were built in OriginPro version 2016 SR0 b9.3.226 (OriginLab Corporation, Northampton, MA, USA).

## Results

### Part I. Quantification of arterial trees on histological sections

#### Means of linear sizes and ratios are not applicable for arterial morphometry on tissue sections

TAs from multiple organs in normal rats were first analyzed using the conventional approach with measurements of mean ED, ID, WTh and WLR (**Table 1**).

**Table 1:** Averaged linear dimensions and wall-to-lumen ratio for terminal arteries with ED of 10–50 μm in different organs.

Organ	ED, μm	ID, μm	WTh,μm	WLR, %
**Liver** (N = 63) CV	21.6±1.4 54%	8.8±0.8** 69%	6.4±0.4 48%	98.6±7.2** 58%
**Adrenal** (N = 61) CV	22.0±1.1 40%	8.7±0.5 41%	6.6±0.4 52%	81.9±5.1 48%
**Kidney** (N = 148) CV	32.7±0.6 24%	13.3±0.4 33%	9.7±0.2 24%	78.9±2.2 35%
**Skin** (N = 225) CV	20.1±0.6 48%	8.5±0.3 52%	5.8±0.2 52%	75.3±1.9 37%
**Skeletal muscle** (N = 98) CV	19.7±0.7* 37%	8.4±0.3* 37%	5.6±0.2* 45%	70.8±3.1 43%
**Bronchial arteries** (N = 50) CV	37.1±1.3 24%	18.4±1.2 48%	9.4±0.4 32%	68.7±7.5 77%
**Heart** (N = 115) CV	26.6±0.9 40%	12.7±0.6 52%	6.9±0.3 42%	63.6±3.0 50%
**Spleen** (N = 203) CV	17.7±0.5 42%	8.3±0.3 51%	4.7±0.1 38%	61.6±1.3 30%
**Small Intestine** (N = 55) CV	18.9±1. 42%	9.5±0.8 65%	4.7±0.2 38%	57.2±3.0 39%
**Stomach** (N = 90) CV	24.9±1.0 41%	12.7±0.6 46%	6.1±0.3 54%	53.5±3.0 54%
**Brain** (N = 144) CV	23.4±0.6 32%	12.7±0.4 40%	5.4±0.2 35%	45.6±1.7 46%
**Pulmonary arteries** (N = 66) CV	29.2±1.3 35%	20.9±1.0 41%	4.1±0.2 34%	21.9±1.1 42%

Data are mean ± SEM. ED–external diameter; ID–internal diameter; WTh–wall thickness; WLR–wall-to-lumen ratio; CV—coefficient of variation; N—number of measured arteries

*P<0.01 vs the bronchial arteries

**P<0.001 vs the brain. Organs are displayed in order of descending WLR values.

**Fig 1:**
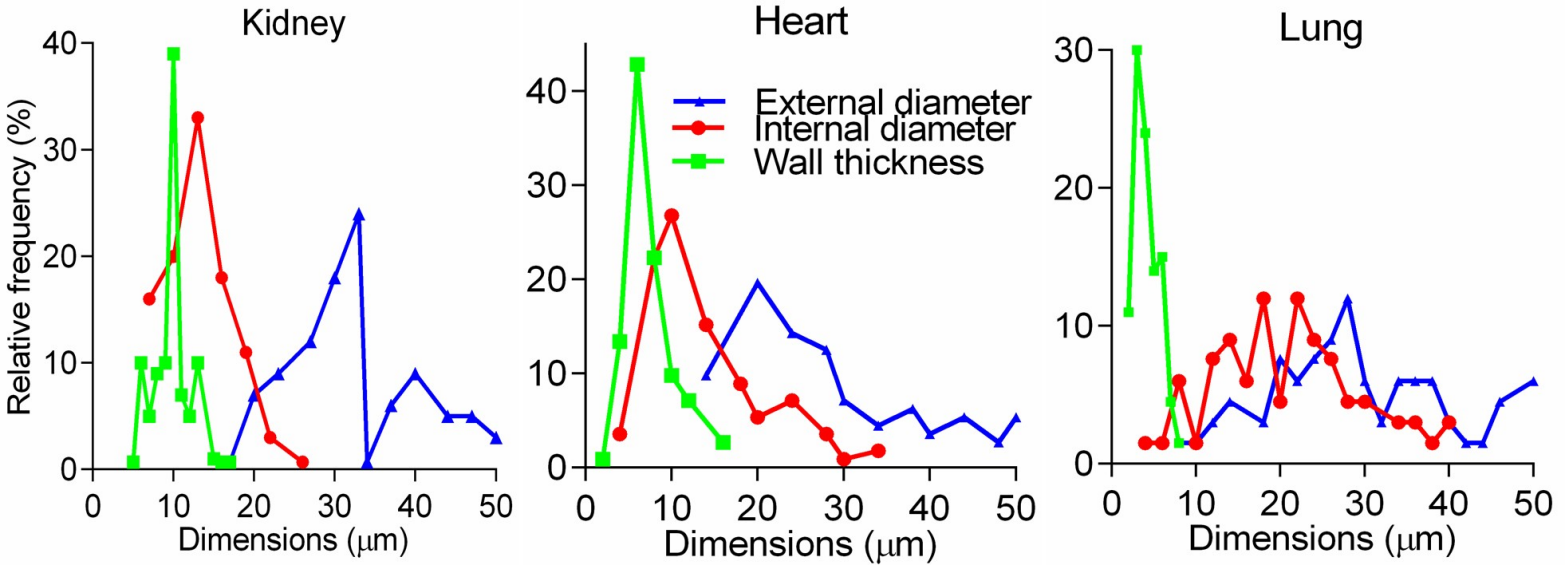
Detailed statistical analysis of primary data. Histograms for the external diameter, internal diameter, and wall thickness in the kidney, heart and pulmonary arteries, used to calculate statistics in **Table 1**, demonstrated irregular frequencies and profound asymmetry.

All values were obtained within the same arterial ED range of 10–50 μm, since larger or smaller arteries were difficult to find in sufficient numbers in tissue samples. TAs in each organ were characterized by distinct average linear dimensions and WLRs. For instance, although bronchial and skeletal muscle arteries demonstrated similar WLRs, their EDs, IDs and WThs differed (P < 0.01). The WLRs for brain arteries were lower but IDs larger than in liver arteries (P<0.001), although EDs and WThs were similar between these two organs. Importantly, coefficient of variation of arterial dimensions were relatively high, varying between 40–70%, indicating their significant diversity[31]. Accordingly, individual histograms for parameter distribution in each organ were prepared. Histograms for ED, ID, and WTh demonstrated considerable variability, without a Gaussian distribution (**Fig 1**). Indeed, as shown in **S1 Table**, the extent of variability for ED, ID and WLR measures did not pass conventional statistical tests for normality.

**Fig 2:**
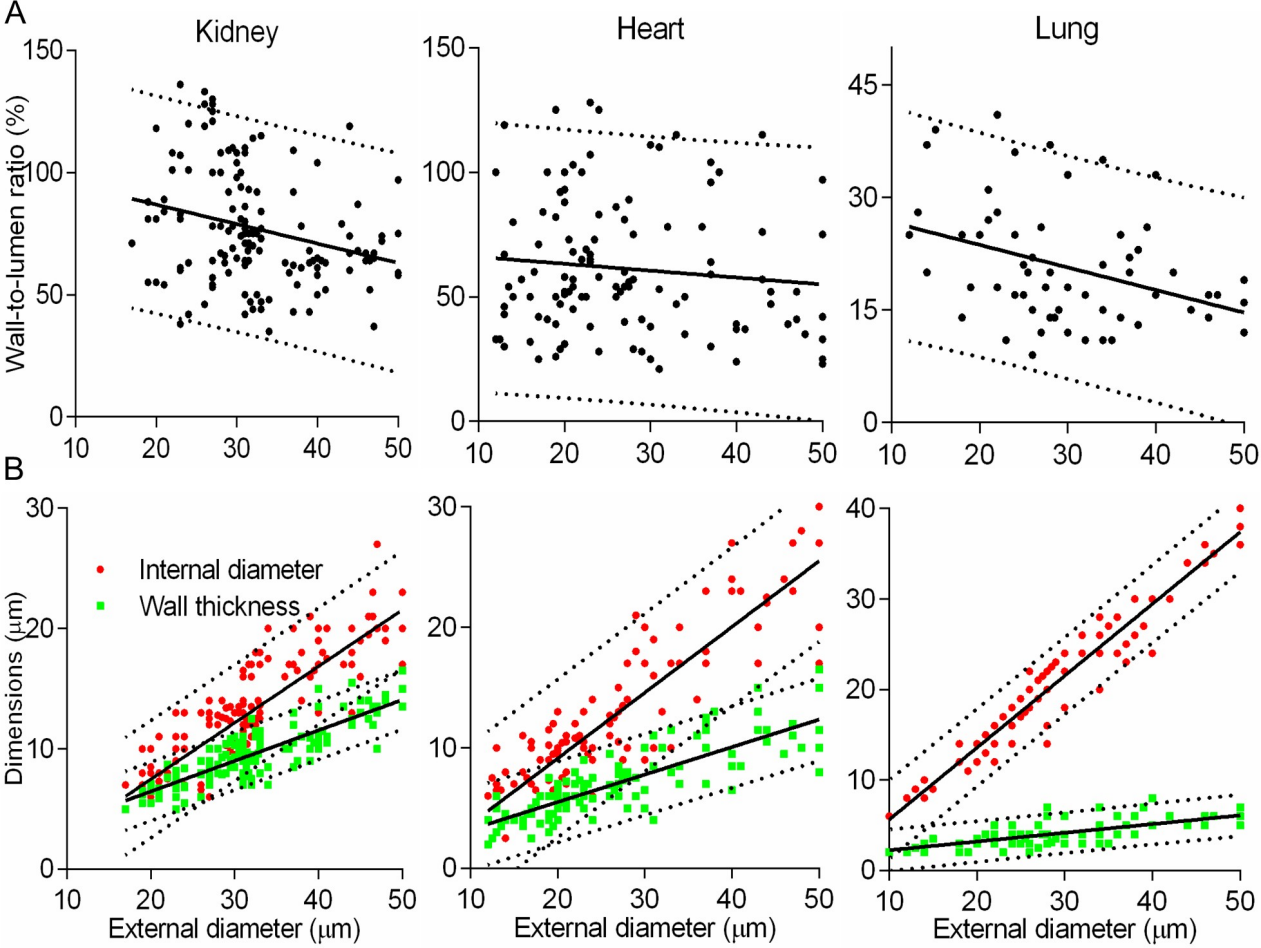
Detailed statistical analysis of primary data. (A) Wall-to-lumen ratio scatterplots were irregular with low r^2^ (solid lines), and wide 95% prediction bands (dashed lines). Corresponding statistics are shown in **Table 1**. (B) Scatterplots of primary data displayed certain incremental change in arterial dimensions. Coefficients r^2^ were moderate, and the pulmonary arteries had the best fitting value.

Our detailed analysis (**Table 1, Fig 1, S1 Table**) indicates that means have little statistical value with regards to such measurements[31]. Even a small range of EDs (10–50μm) for TAs in all organs was associated with an abnormal distribution and high variability, while WLRs were not constant as expected (**Fig 2A)**. Furthermore, increasing numbers of measurements to normalize distribution and reduce variability were not effective for tapering branching objects[30].

#### Application of short intervals provides precise complex profiles and linear regression equations

**Fig 3:**
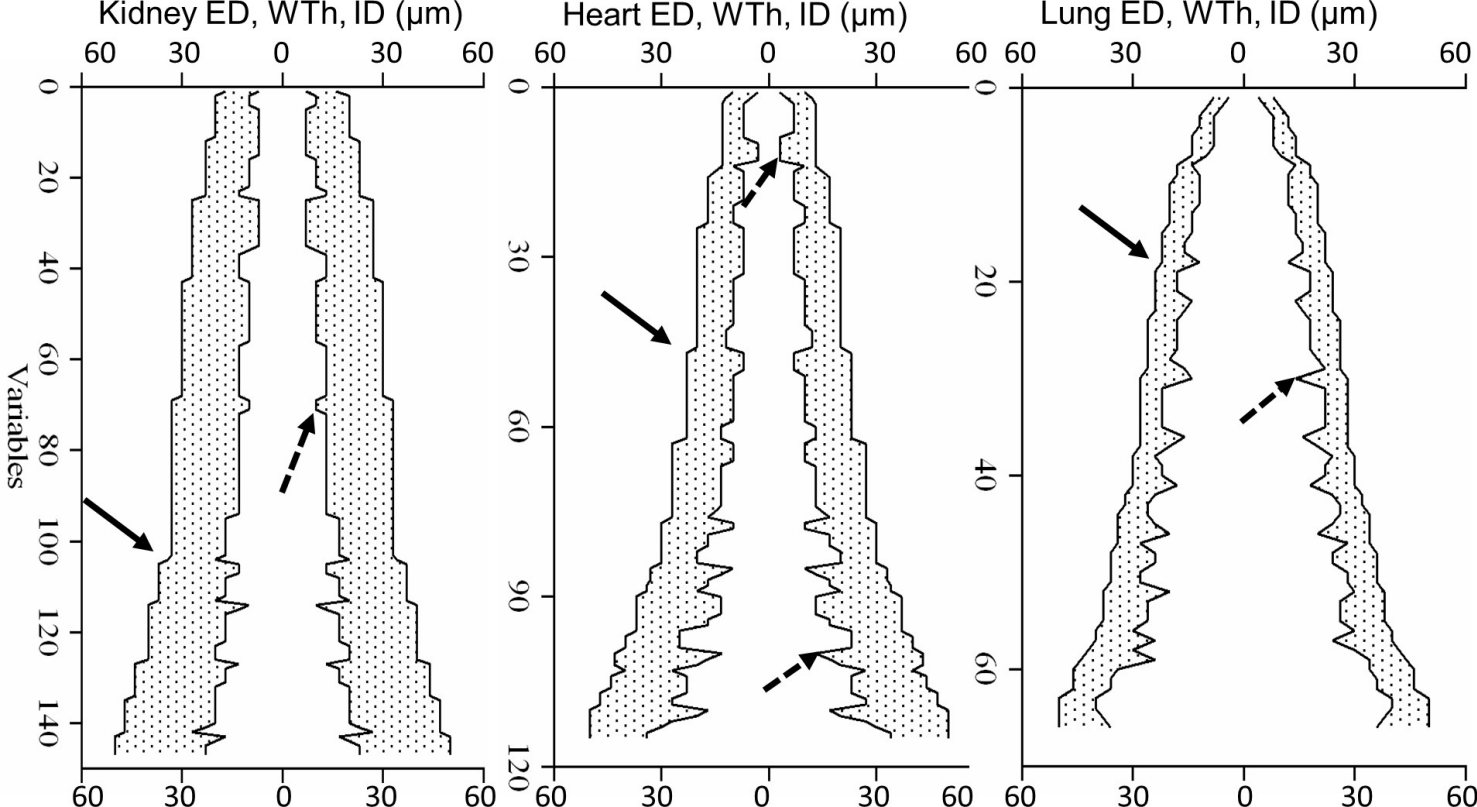
Analysis of primary data with complex profiles. Primary measurement for the kidney, heart, and pulmonary arteries were arranged in the complex profiles. Axis X–the bidirectional common scale for the external diameter (ED, outer contours), internal diameter (ID, inner contours), wall thickness (WTh, shaded regions); axis Y represents the number of measured arteries (variables) in order of ascending ED. Steps in the outer contours (solid arrows) reflected a minimal division on an eyepiece micrometer. Outliers along the internal contours (dashed arrows) corresponded to wider or narrower ID, compared to neighboring ED values.

For vessels with EDs in the range of 10–50 μm, there was a trend towards dependence of ID and WTh on ED, although variability was substantial (**Fig 2B**). We therefore assessed primary data with a complex profile method, which is widely used in cartography, thermodynamics, and engineering[32],[33]. In this technique, a contour line is drawn as a function of two variables, where the gradient of function is perpendicular to the contour isoline, thus representing more than two dimensions on a two-dimensional (2D) graph. To be applied to measuring arteries, each arterial circle on a histological section is first characterized by three values: ED, ID, and WTh. Then all measured vascular circles were arranged in a row in order of ascending ED, and numbered from 1 to N, where N is the total number of measured arterial rings. Subsequently, each number was plotted on the y-axis, while its corresponding ED, ID and WTh were plotted on a bidirectional x-axis (**Fig 3**).

The same primary data (**Table 1, Figs 1 and 2)**, represented in this way, revealed a regular hemodynamic structure. Staircase steps of the external contours were relatively small and regular since a conventional microscope with a 40x objective provided a minimal division value of ~2 μm to the eyepiece micrometer, so that each ED was rounded off to ± 2 μm. Spikes of internal contours were larger and quite irregular corresponding to some arterial segments with similar ED but different ID and/or WTh. Presumably, those outliers represented subsets among arterial segments, branching at different hydrodynamic points. Outliers are not evident in primary data plots to be removed (**Fig 2**). Moreover, a conventionally recommended increase in number of measurements[31] simply increases the number of outliers. To make outliers more evident, instead of conventional averaging of the data for the entire range of 10–50 μm, the ED was divided into short regular intervals. We tested different intervals, and found that the interval of 5 μm was optimal for all organs.

**Fig 4:**
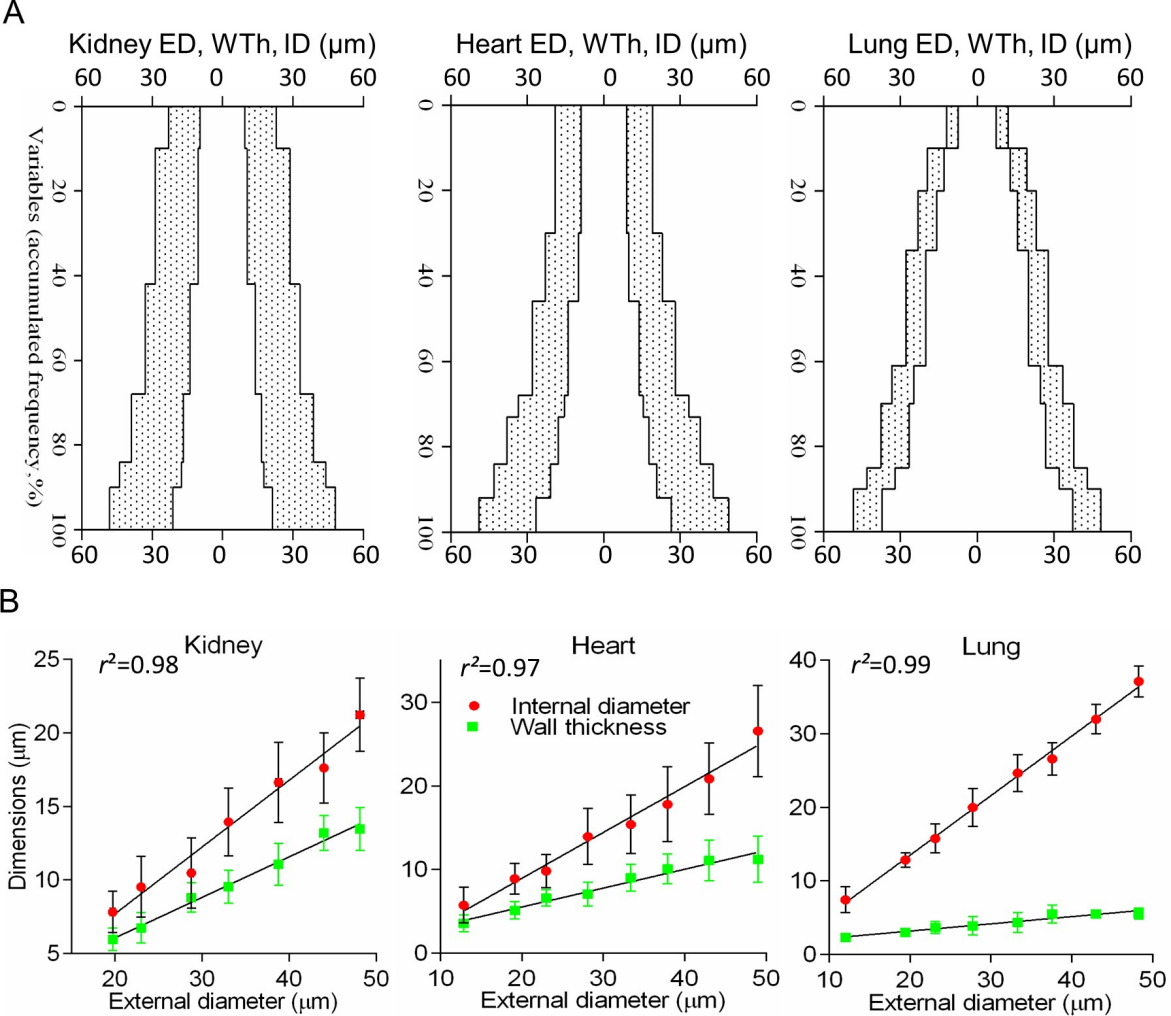
Application of the short fixed intervals significantly improved primary data analysis. (A) Complex profiles were built from the accumulated frequencies (axis Y) of the means of the external diameter (ED), internal diameter (ID), and wall thickness (WTh) for 5-μm ED intervals. In contrast to scatterplots in Fig 2B, terminal arteries exhibited distinctive tapering patterns in organs. Complementary graphs are in S1 Fig. (B) Mean values of ID and WTh for the regular 5-μm ED intervals revealed robust linear regression equations (P<0.001). Complementary graphs are in S2 Fig. Points on the lines are mean ± SD for 5-μm ED intervals.

When the complex profiles had been drawn not from every measured value, but from the mean interval values, they demonstrated organ-specific gradients in tapering (**Fig 4A, S1 Fig**). Furthermore, in contrast to data depicted in **Fig 2B,** if outliers had been removed, the means of short intervals revealed very tight linear regression between both lumen and wall thickness and vessel caliber in kidney, heart and pulmonary vessels, and in other organs as well (**Fig 4B, S2 Fig).** All acquired equations demonstrated goodness of fit coefficients *r*^*2*^ between 0.8–0.9 (P <0.0001), with few exceptions for WTh in the brain and bronchial arteries (**S2 Table**).

To decide whether linear regression equations for TAs were organ-specific, their slopes and intercepts were compared (**S3 Fig)**. Equations for pulmonary, bronchial, adrenal, stomach and skeletal muscle TAs were distinct (P<0.0001). Heart and spleen TAs had similar equations, as did brain and intestine, and kidney, liver, and skin.

#### Hemodynamic significance of morphometry data

**Fig 5:**
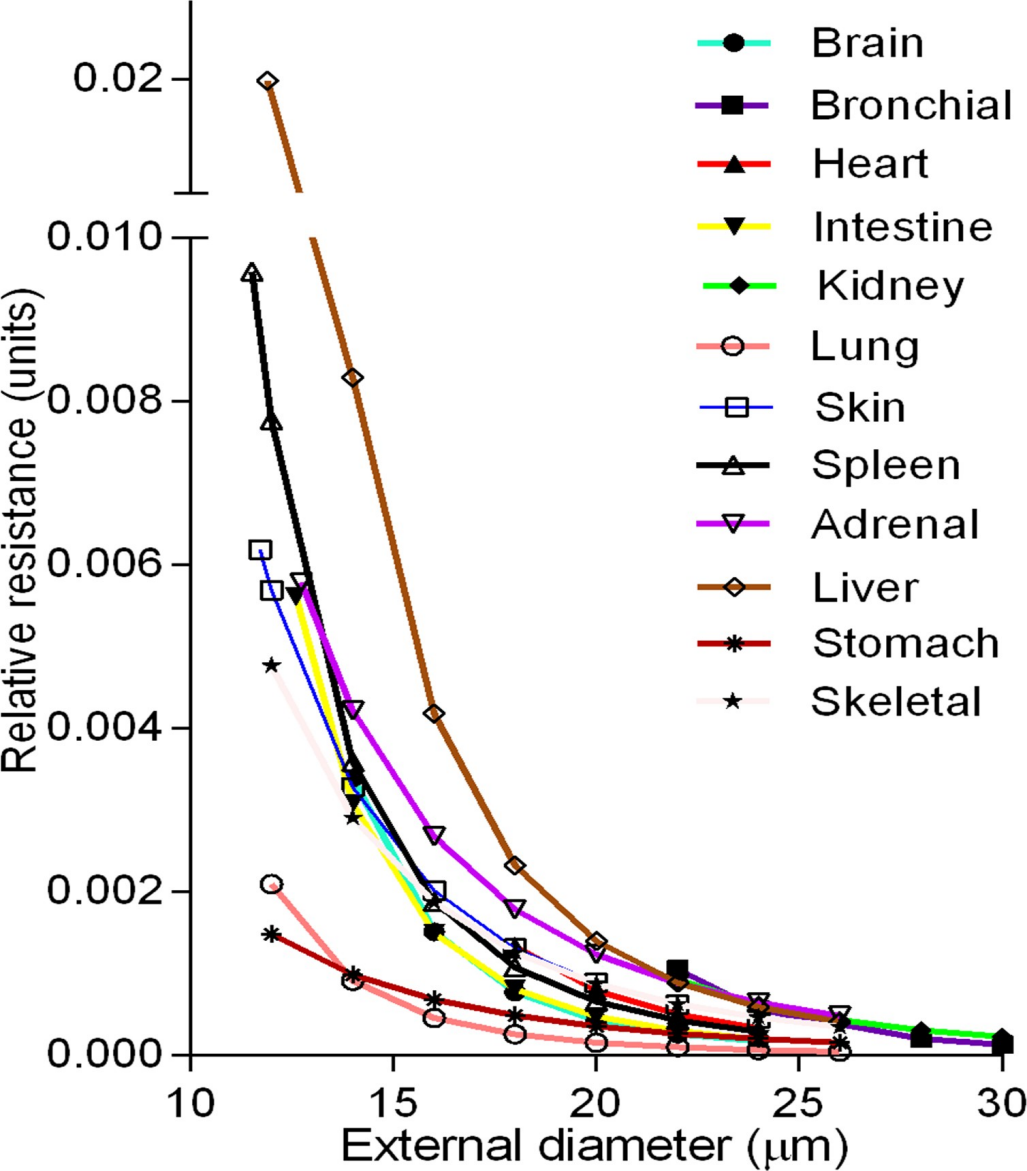
Comparative analysis of the relative resistance in different organs. Relative resistance curves were calculated from the regression equations in **S2 Table**. The liver and spleen had the largest values (P<0.001), while the pulmonary and stomach arteries—the lowest (P<0.001).

Reliable linear regressions also validated an assessment of relative resistances (RR) that was calculated using Poiseuille’s relationship RR = 1/πr^4^, as recommended[34],[35],[36], assuming constant viscosity and length, and where **r** is the radius of the vessel lumen. RRs for 5 μm regular intervals across multiple organs are depicted in **Fig 5.** The highest values are in liver, and lowest in pulmonary arteries, rising exponentially in the smaller segments, since blood flow is proportional to the fourth power of the luminal radius.

**Fig 6:**
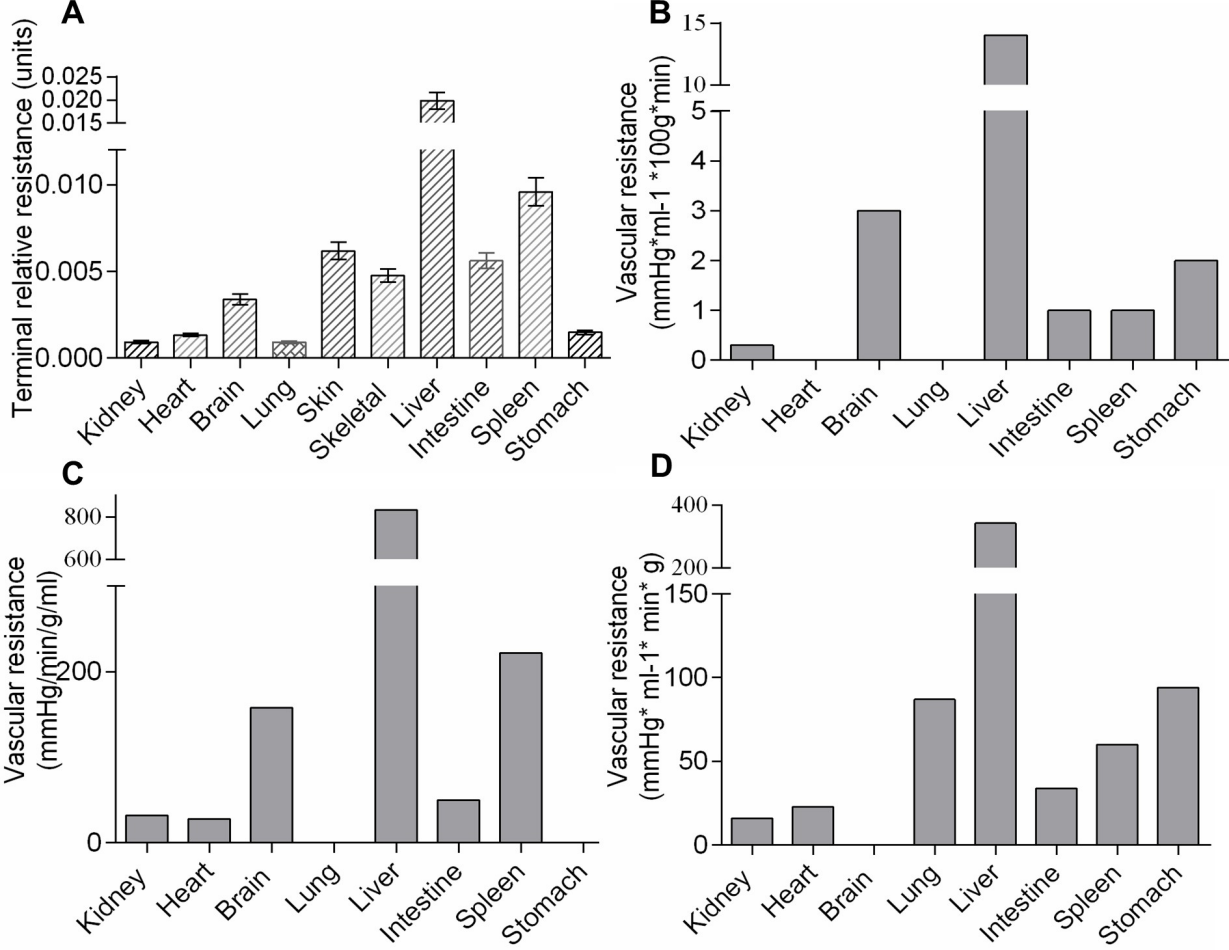
Correspondence between morphometric and hemodynamic parameters. (A) TRR demonstrated similarity in organ-to-organ ratio with vascular resistances acquired with physiological methods: modified from[37] (B); [38](C);[39] (D). Data on (A) are mean ±SEM, on (B-D)–mean values.

Accordingly, terminal relative resistance (TRR) would estimate the resistance for the smallest vessels of ED = 10–20 μm that have the largest accumulated frequency in complex profiles (**Fig 6A**).

Since the complex profiles reveal the average numbers of arterial branches of defined lumen diameters in each organ, prior to the capillary bed, it would be reasonable to measure terminal capacity (TC) in each organ, as the sum of the internal volume of each segment multiplied by its frequency:

$$\mathrm{TC}=\sum (\pi {*(IDi)}^{2}/4)i\%$$ (1)

**Fig 7:**
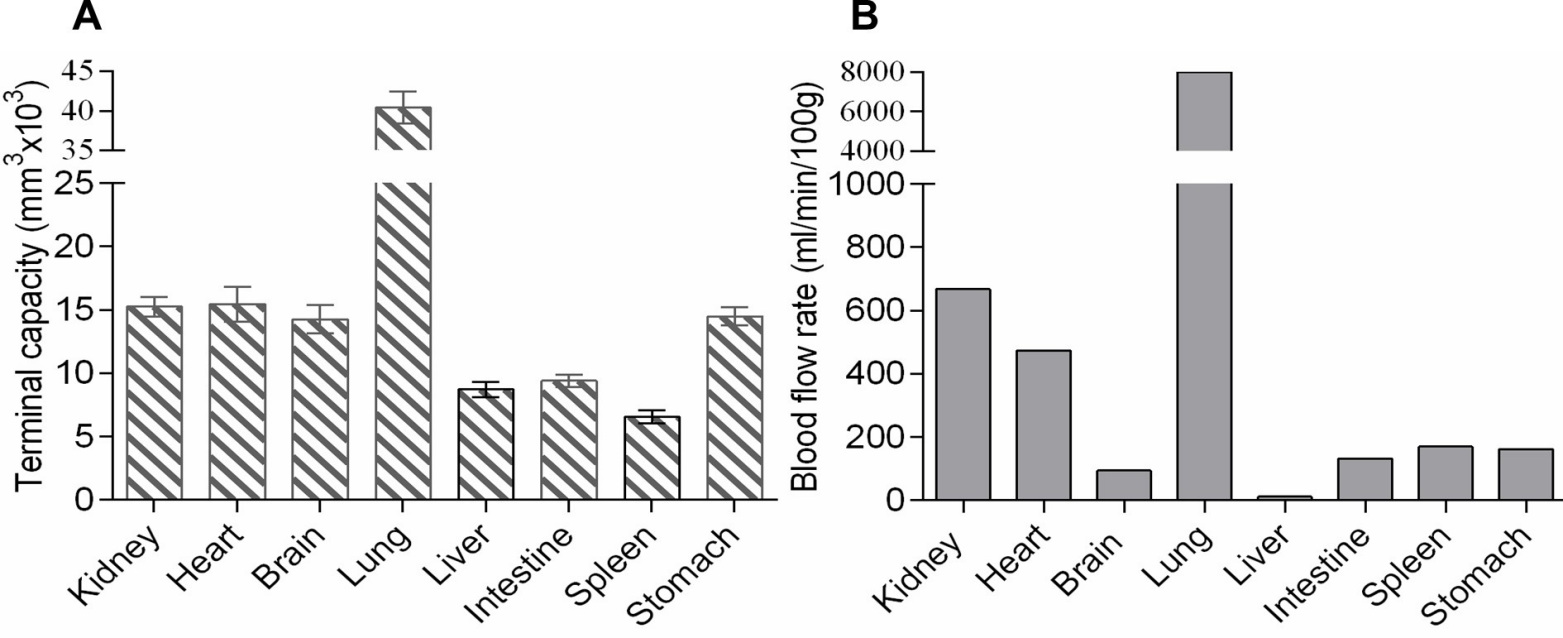
Correspondence between morphometric and hemodynamic parameters. (A) TC values were in good agreement with organ blood flow rates, estimated by a physiological method[40] (B). Data on (A) are mean ±SEM, on (B)–mean values.

Where **ID***i* is the mean ID of each interval, calculated from the linear regression equation; *i*% -the frequency of that interval in the complex profile (**Fig 7A).**

#### The method enables comparison between species

**Fig 8:**
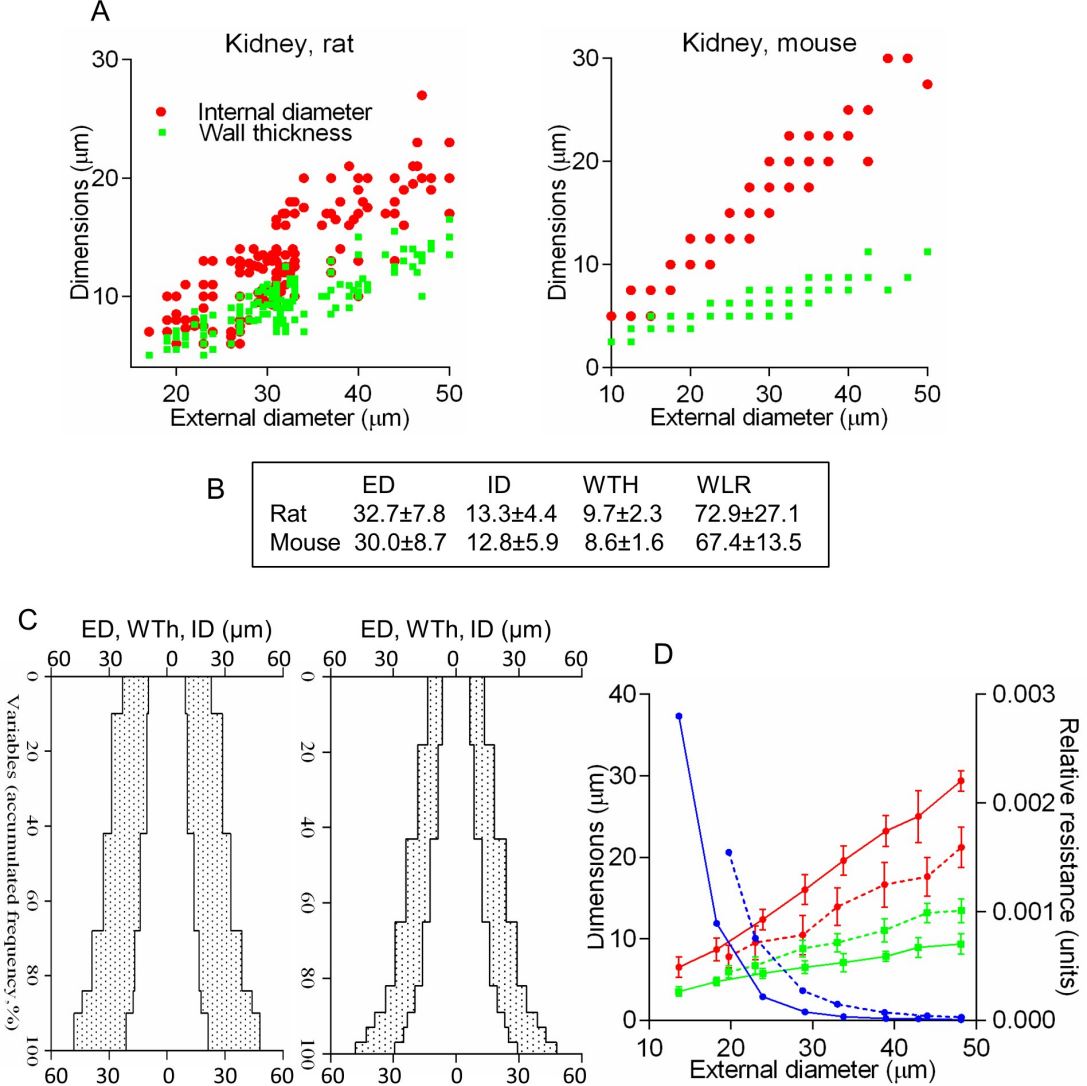
The algorithm enables comparison between species. (A) Scatterplots for the external diameter (ED), internal diameter (ID), and wall thickness (WTh) for the rat and mouse. (B) Mean values from scatterplots revealed no difference (P>0.40), including wall-to-lumen ratio (WLR). The corresponding complex profiles (C), and regression equations with the relative resistance curves (D) were very distinctive (P<0.001) for the rat (dashed line) vs mouse (solid line).

Using this approach, we compared TAs between normal rat and mouse. While averaged dimensions demonstrate no substantial differences, the complex profiles, linear regression equations and RR curves revealed specific patterns for each species (**Fig 8**).

### Part II. Recognition of remodeling patterns in hypertensive rats on histological sections

#### Conventional measurements exhibit different remodeling patterns in hypertensive rats

At 60 days, the systolic blood pressure in the sham-operated and hypertensive rats was 115±6 mmHg and 217±21 mmHg, respectively (P<0.001). Morphometry data from multiple organs were first analyzed using conventional measures of mean ED, ID and WTh for the arterial ED interval of 10–50 μm. 1K1C hypertension significantly affected TAs in every organ (**Table 2**).

**Table 2:** The spectrum of remodeling variants in terminal arteries of ED 10–50 μm.

Organ	Sham rats			1K1C rats			WLR	MCSA	NC
	ED, μm	ID, μm	WTh, μm	ED, μm	ID, μm	WTh, μm			
**Brain**	23±7	13±4	5±2	19±8**↓	7±4**↓	6±2 *↑	↑	↓	**15**
**Kidney**	33±8	13±4	10±2	30±9**↓	10±4**↓	10±3 ↔	↑	↓	**14**
**Heart**	27±9	13±6	7±3	27±10 ↔	11±4**↓	8±3*↑	↑	↑	**9**
**Pulmo-** **nary**	29±8	21±8	4±1	33±9**↑	22±6 ↔	6±1**↑	↑	↑	**2**
**Skin**	20±9	8±4	6±3	23±9**↑	8±3 ↔	7±3**↑	↑	↑	**2**
**Skeletal** **muscle**	20±7	9±4	5±3	21±10 ↔	8±3**↓	6±3 **↑	↑	↑	**9**
**Bronchial**	37±9	18±7	9±1	32±10**↓	13±6**↓	10±2**↑	↑	↓	**15**
**Stomach**	25±7	13±5	6±3	27±11*↑	10±5**↓	8±3**↑	↑	↑	**1**
**Intestine**	19±8	9±6	5±1	24±10**↑	9±4 ↔	7±3**↑	↑	↑	**2**
**Adrenal**	22±8	9±3	6±2	24±11**↑	14±5**↑	5±3*↓	↓	↓	**4**
**Liver**	22±9	8±4	6±2	22±12 ↔	7±4 ↔	7±3 ↔	↔	↔	**-**

Data are mean ± SD. ED–external diameter; ID–internal diameter; WTh–wall thickness; WLR–wall-to-lumen ratio; MCSA–media cross sectional area; NC–numerical classification. (↑) (↓) (=) indicate increase, decrease or no change respectively vs control values.

*P<0.05

**P<0.01.

**Fig 9:**
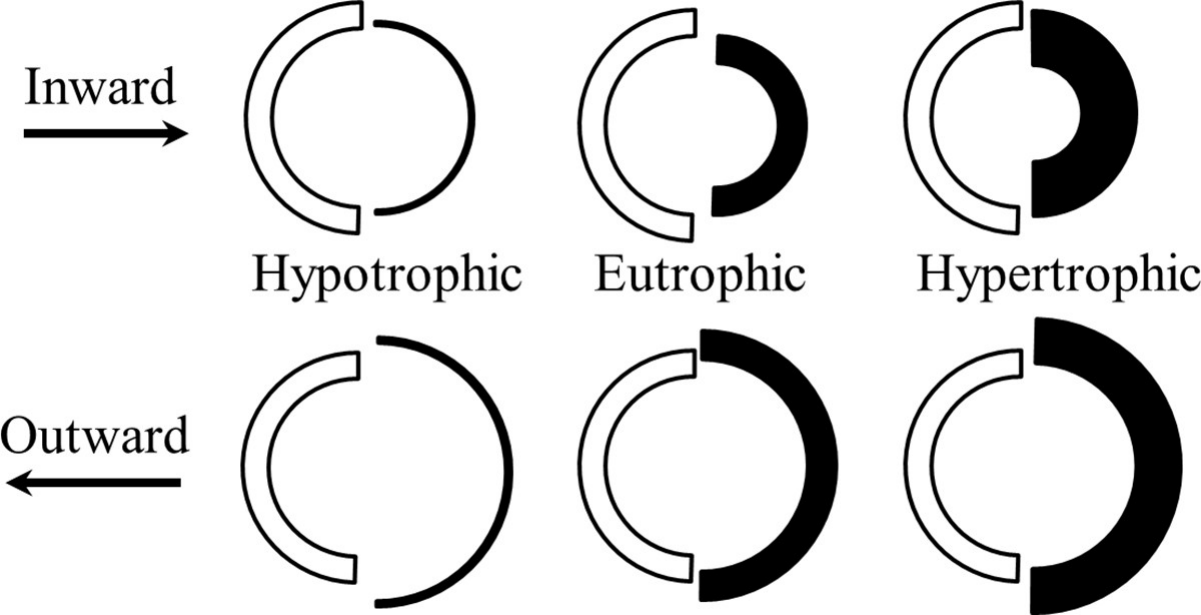
The descriptive conventional classification. The ‘inward’ or ‘outward’ means reduction or increase in lumen; the ‘hypertrophic,’ ‘eutrophic,’and ‘hypotrophic’ indicates increase, no change, or decrease in wall cross-sectional area, respectively.

However, the obtained remodeling varieties did not fit into conventional definitions[10] (**Fig 9**). The classification considers ‘inward’ or ‘outward’ as only simultaneous ED-ID decrease or increase. The brain, bronchial and kidney should be named ‘inward hypotrophic’ due to reduced ED, ID and MCSA, but that designation does not recognize increased or stable WTh. The classification has no ‘inward-outward hypertrophic’ type that appeared in data for the stomach. The heart and skeletal muscle arterial remodeling should be defined as ‘inward’ because mean IDs were reduced, but constant mean EDs are not recognised, and there is no ‘inward-only hypertrophic’ variant. The same premise applies to constant IDs in the pulmonary, skin, and intestinal arteries that should be classified as ‘outward-only hypertrophic.’ For unknown reasons, remodeling has been considered only in the context of maintaining a constant MCSA, and not constant WTh[10]. Averaged linear dimensions demonstrated a high degree of variability and abnormal distribution in control and hypertensive animals (**S4 Fig**). Accordingly, conventional averaging of dimensions led to the designation of diverse remodeling patterns.

#### The conventional classification is unable to categorize remodeling variants

**Fig 10:**
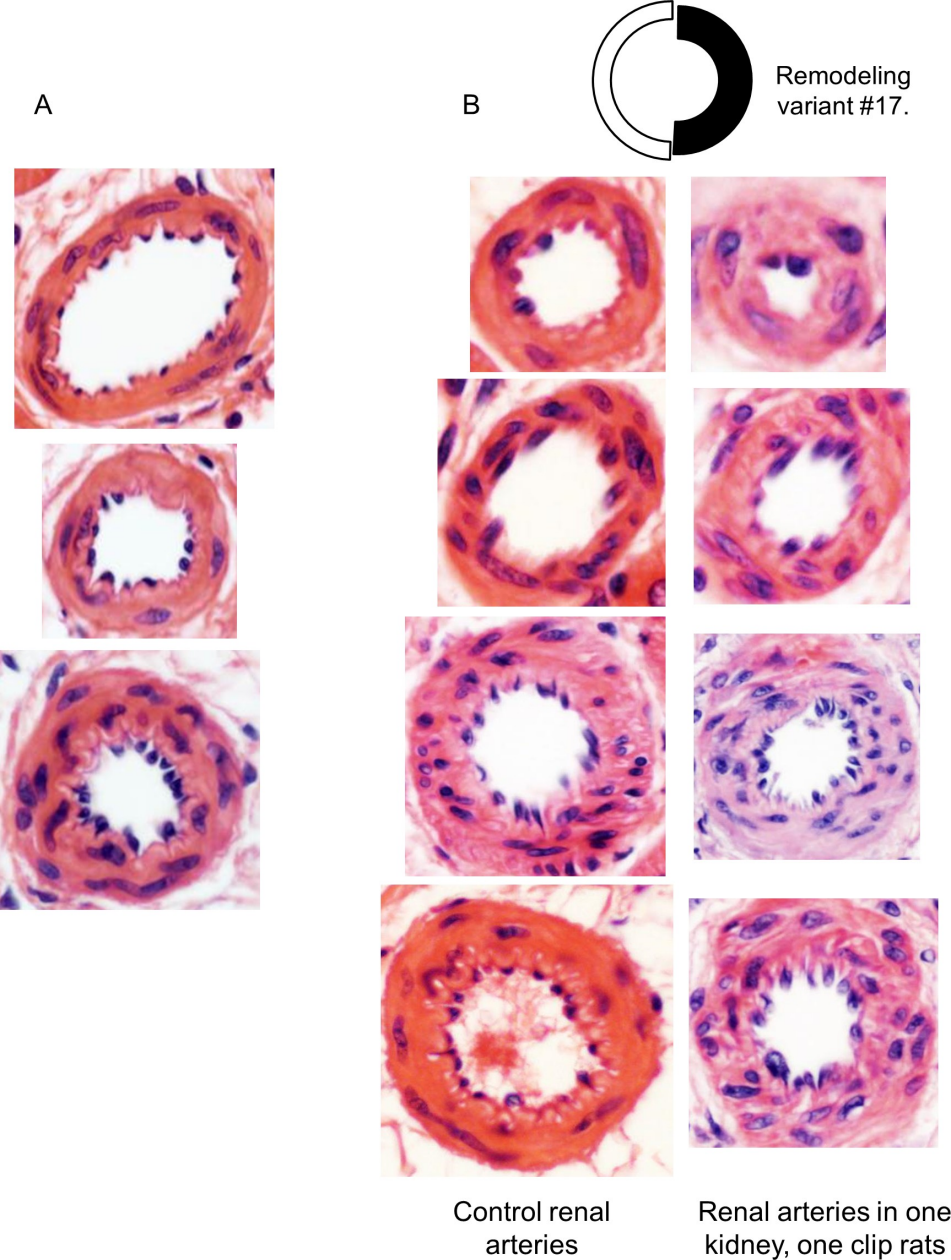
The conundrum in arterial remodeling assessment. (A) Rat arteries in hypertensive rats. Each artery could be an image of another one before or after remodeling: there is no a structure or phenotype marker indicating its previous dimensions. (B) Identification of a remodeling variant enables comparative analysis in arteries of a different range. Hematoxylin-eosin, x400.

The analysis of the annular rings in histological sections represents a conundrum, since arteries have no ‘reference points’ from which they have been modified to assume a remodeled shape (**Fig 10A**).

**Fig 11:**
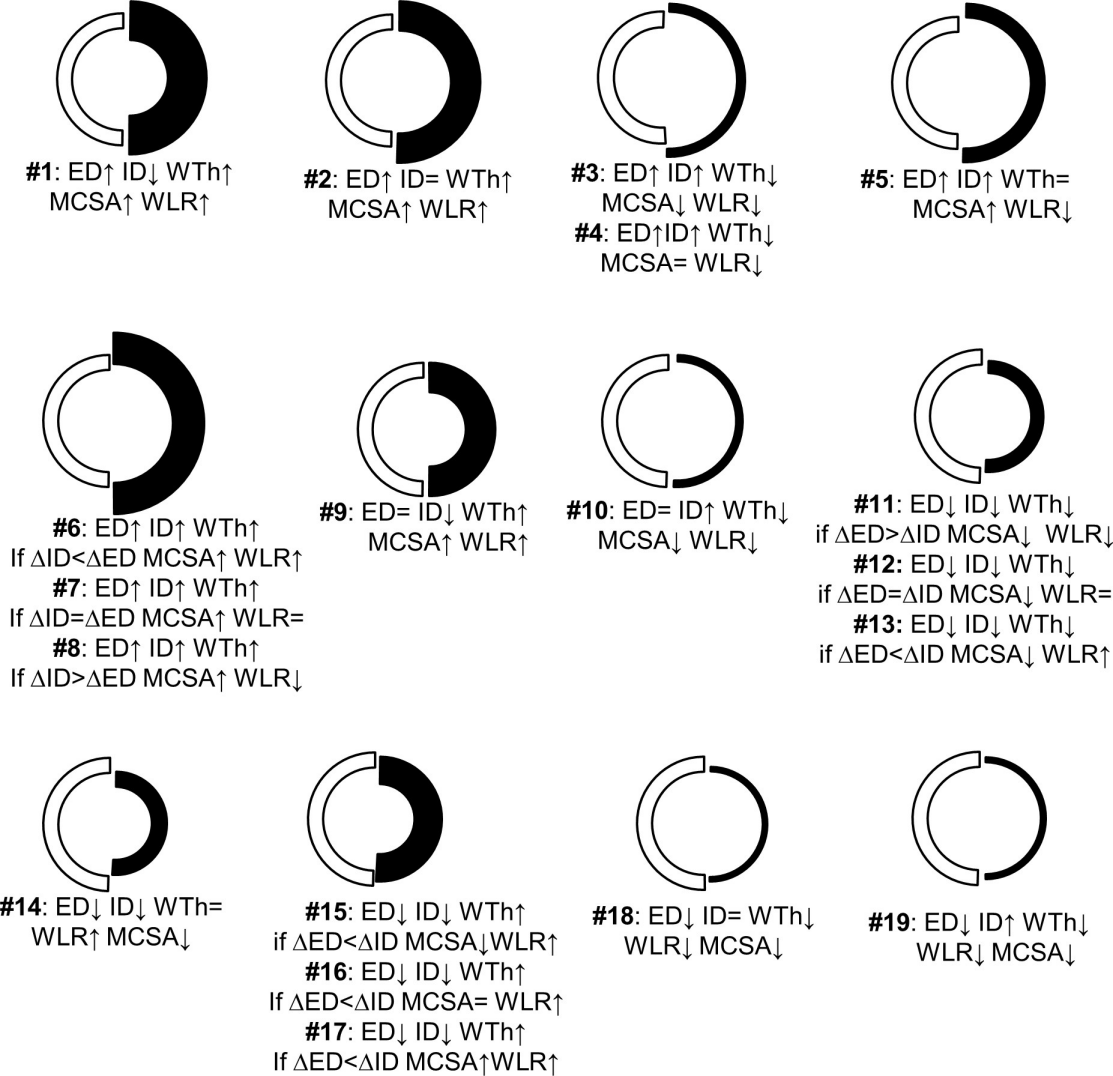
The predicted remodeling variants. The increased external diameter (ED, ↑) will develop the variants #1–8. Conformation with stable ED (=) could follow only through the variants #9 and 10. Reduction in ED (↓) could follow the variants #11–19. ID–internal diameter; WTh–wall thickness; MCSA–media cross sectional area; WLR–wall-to-lumen ratio. White semicircles–normal, black–predicted remodeling variants.

In order to precisely categorize remodeling variants in consideration of the conventional designations of inward-outward and hypertrophic-hypotrophic (**Fig 9**), possible conformations of a blood vessel were modeled with the approximation of arteries as thick-wall cylinders capable of changing ED and ID independently. In a simulation procedure, each variant was considered distinct if a value for only one dimension in the set of five (ED, ID, WTh, MCSA, WLR) was different from the value for another set. The numerical modeling (**Fig 11**) revealed not six, as empirically suggested, but nineteen variants of arterial remodeling. For example, increasing ED produces eight (#1–8) possible variants by combination of changing ID, WTh and MCSA in different directions. A constant ED results in two variants (#9, 10), and decrease in ED generates nine variants (#11–19). The variants #6–8, 11–13 and 15–17 are possible because not only the direction but even gradients of change between ED and ID could elicit unique remodeling patterns, which were impossible to classify with conventional definitions. Therefore, the conventional term “hypertrophic” would equally apply to the variants #1, 2, 6–9, and 15–17. The term “outward” would define simultaneously the variants #1–8. The term “eutrophic” would equally apply to the variants #5, 14 and 16, while “hypotrophic” could be true for #3, 4, 10–13, 18, and 19.

According to this numerical classification, in 1K1C rats TAs demonstrated seven remodeling variants across organs (**Table 2)**. Studies using either random sections (**S3 Table**) or *in vitro* myography (**S4 Table)** also demonstrate variable multidirectional remodeling patterns.

To decide whether remodeling varieties truly exist or are derived from conventional averaging, we tested methods recognizing that arteries follow one of nineteen predicted variants.

#### Remodeling patterns are not identified on 2D graphs

**Fig 12:**
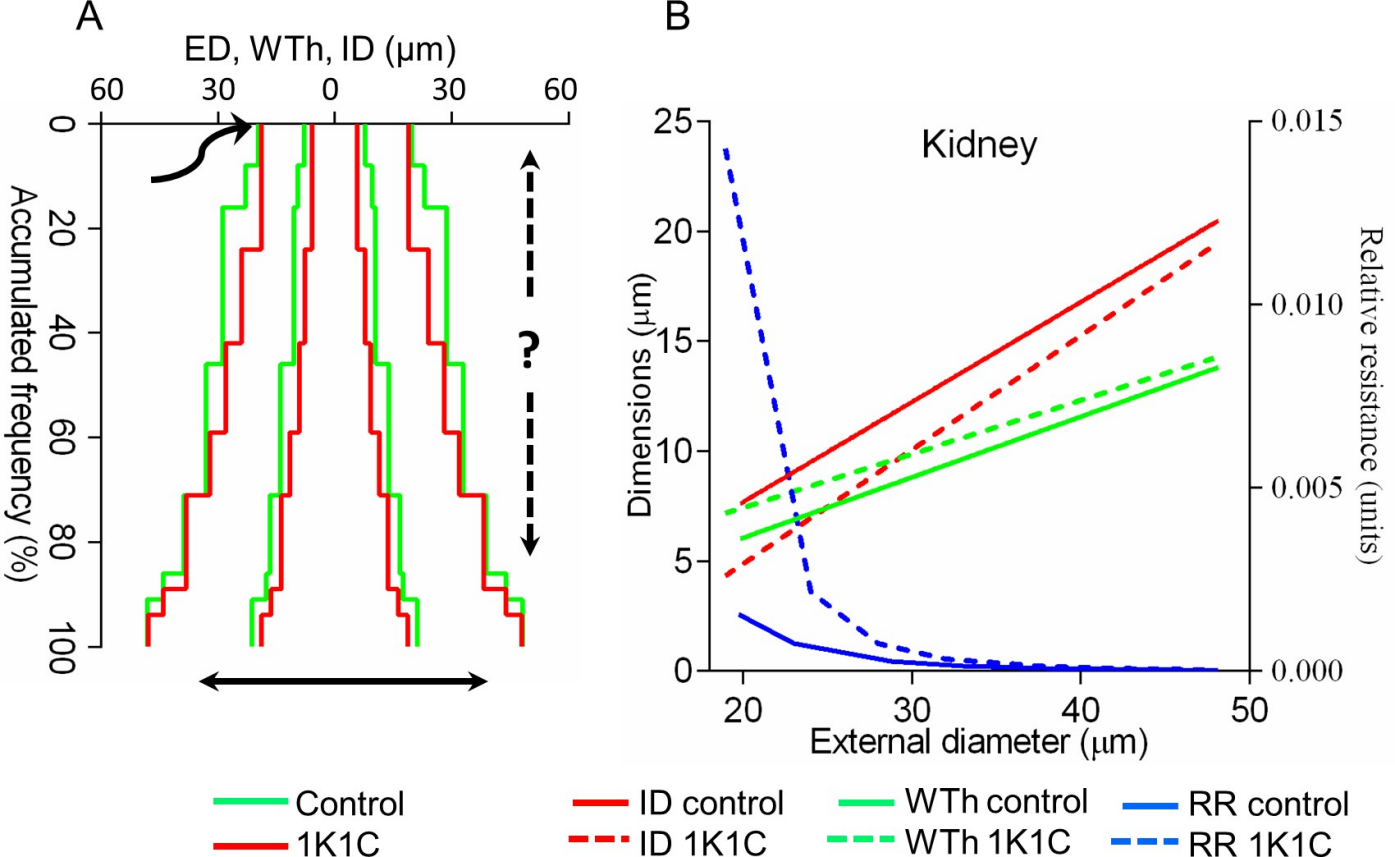
Considerations for arterial remodeling assessment. (A) The profile of hypertensive (red) renal arteries was superimposed on the control (green). The starting point (the curved arrow) is uncertain: inward–outward remodeling (the solid double arrow) is well known, while proximal-distal shifting (the dashed arrow) is unacknowledged. Uneven accumulated frequencies displaced interval values irregularly. Complementary graphs are in **S5 Fig**. (B) Linear regression lines of renal terminal arteries demonstrated the decreased internal diameter (ID) and increased wall thickness (WTh) in hypertension. The slopes were different (P<0.0001). ED–external diameter; RR–relative resistance. Complementary graphs are in **S6 Fig**.

As in control rats, primary measurements were organized in 5 μm intervals, the mean value for each interval was calculated, and the complex profile and linear regression equations built from the means of intervals (**S5 Table**). Data analysis revealed that comparison of complex profiles could not be used to identify remodeling variants because it was complicated by the existence of uncertain starting points for superimposition of a hypertensive graph on the control one (**Fig 12A, S5 Fig**). There are no data indicating how vessel remodeling progresses in a length-wise fashion, except for the presence of increased vessel tortuosity[26],[34]. While the common starting points were unclear, varying accumulated frequencies suggested distinct remodeling patterns even in different segments within the same organ (**Fig 12A, S5 Fig**).

Next, we tested if linear regression could identify remodeling patterns. Compared to the complex profiles, the linear regressions demonstrated smaller IDs and bigger WThs for the same ED similarly in all organs, except the kidney and adrenal (**Fig 12B, S6 Fig**).

**Fig 13:**
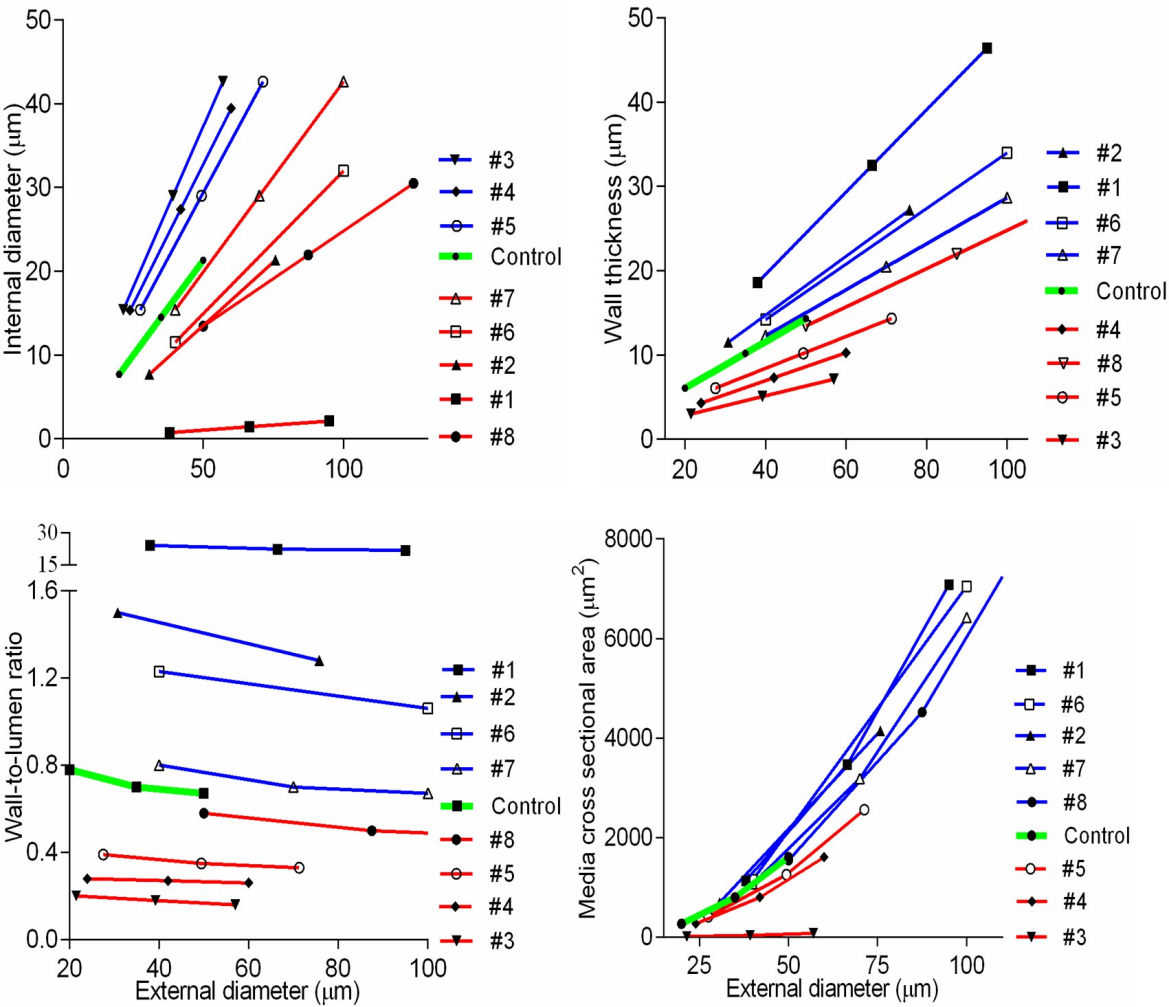
Remodeling variants are not distinguishable in two-dimensional graphs. The remodeling of renal arteries for the variants #1–8 show similar line shifts, that could distinguish only increase or decrease from control but not verify a particular pattern. Complementary graphs are in **S7 Fig**.

To understand how shifts in regression lines could verify remodeling, every predicted variant, for each organ, was simulated from control linear regression equations (**S5 Table**). Simulation was done for: linear sizes including ED, ID, WTh, IP, EP; areas, such as MCSA, LCSA, total area (MCSA+LCSA), and ratios, including WLR, EP/IP, and MCSA/LCSA. For all tested dimensions, the regression line displacement from the control line was similar for as many as 11 variants (**Fig 13, S7 Fig)**. Any linear size, area or their ratio exhibited similar displacement for many different remodeling variants. While the use of linear regression equations can verify the presence of remodeling, the equations are unable to define a specific remodeling pattern. Thus, it was not possible to distinguish arterial spatial conformations on routine 2D graphs.

#### Remodeling patterns are not identified with WLR, RI or GI

Increased or decreased WLR is widely used as the main criteria of ‘inward’ vs ‘outward’ wall conformation[3],[4],[5]. This is not appropriate since WLR could be similarly increased in nine, decreased in eight, or unchanged in three variants (**Fig 11**). Therefore the frequently used WLR is unable to define either wall thickening or lumen narrowing.

Remodeling and growth indices are also regarded as the main parameters to estimate remodeling[22]. The primary data[6] was interpreted by the authors as a combination of hypertrophy and rearrangement of vascular smooth muscle cells. The proposed combination was explained with two formulae. The formula (2) below was based on the first presumption: what would the ID be if hypertensive MCSA (MCSA*hr*) remains equal to normal MCSA (MCSA*n*), i.e. vessels developed the variant #16 (*ID*_*16*_)? The formula (3) below was based on the second presumption: what would the ID be if hypertensive ED (ED*hr*) remains equal to normal ED (ED*n*), i.e. vessels developed the variant #9 (ID_*9*_)? To create the formula (2), the larger MCSA*hr* was ignored in favor of a proposed MCSA*n* = MCSA*hr* to calculate percent of encroached lumen if the variant #16 would occur:

$$\mathrm{Percent}\;\mathrm{of}\;\mathrm{encroached}\;\mathrm{lumen}=\frac{IDn-\sqrt{{EDhr}^{2}-4MCSAn/\pi }}{IDn-IDhr}=\frac{IDn-ID(16)}{IDn-IDhr}$$ (2)

For the formula (3), the smaller ED*hr* was ignored in favor of a proposed ED*n* = ED*hr* to calculate percent of encroached lumen if the variant #9 would occur:

$$\mathrm{Hypertrophy}=\frac{IDn-\sqrt{EDn^{2}-4MCSAhr/\pi }}{IDn-IDhr}=\frac{IDn-ID(9)}{IDn-IDhr}$$ (3)

The primary definition “percent of encroachment lumen” in the first formula was renamed the “remodeling index”, and the second formula for “hypertrophy” was modified to “growth index” by[41]. Unlike the third formula, GI counted true MCSAs:

$$GI=\frac{MCSAhr-MCSAn}{MCSAn}$$ (4)

Accordingly, the formulae were intended to quantify only *a subjective interpretation* of the data as a combination of variants #9 and #16. That interpretation could be valid as any combination between eight variants with diminished ID.

#### A method of 3D-modeling simulation recognizes remodeling type

**Fig 14:**
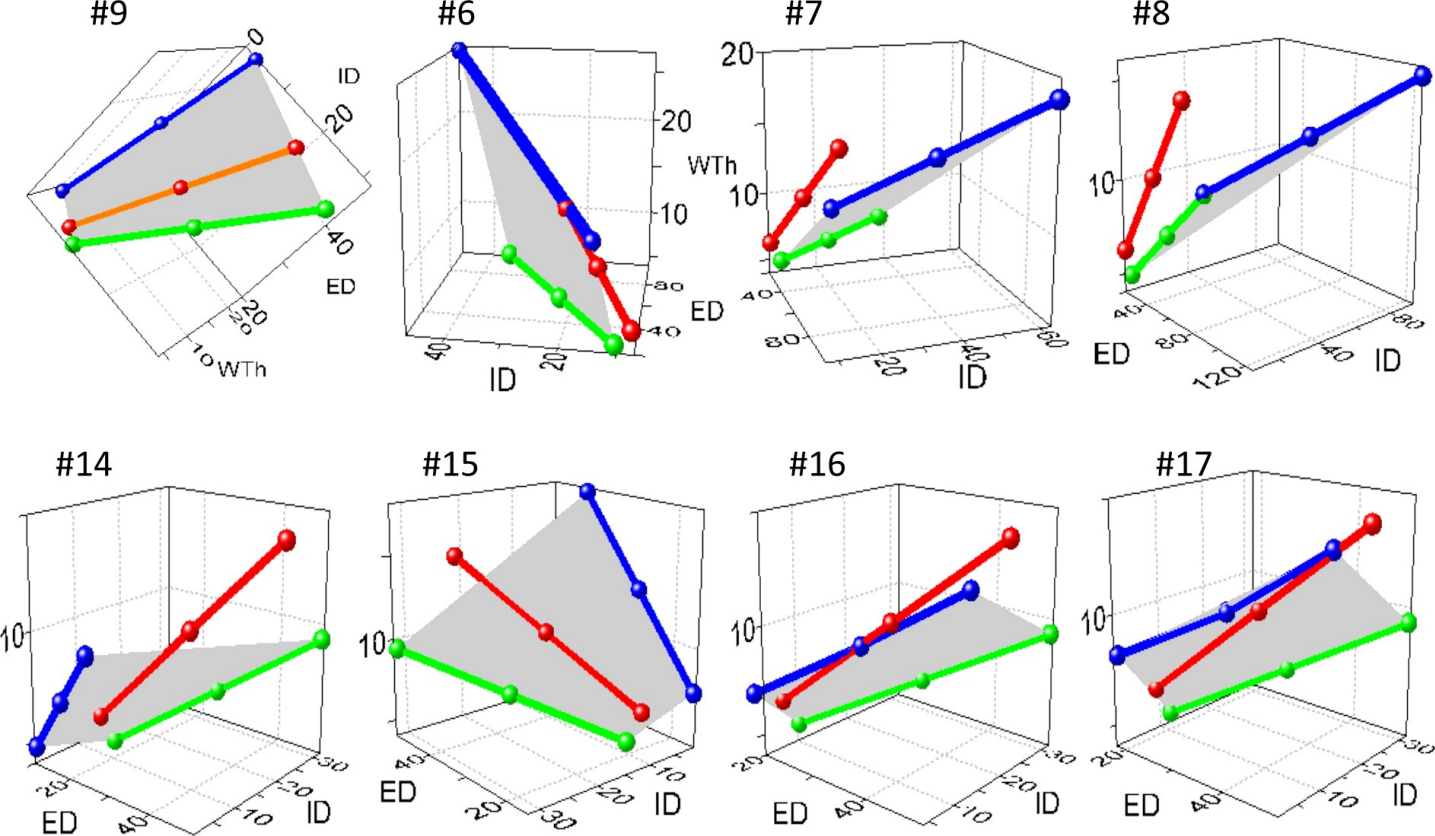
3D modeling simulation for the brain. In 3D graphs, the control equation (green lines) and each predicted variant (blue lines) are connected by the planes (grey) representing sets of all possible values that external diameters (ED), internal diameters (ID), and wall thicknesses (WTh) could acquire during transformation. The red lines represent the regression equation for hypertensive rats (S5 Table). The red lines were not congruent with any of the predicted planes, except for the variant #9. The method readily distinguishes the gradients ΔED, ΔID, and ΔWTh for the variants #6–8 and 15–17 that could only be implied in 2D graphs in Fig 13. Axis X–ED, axis Y–ID, axis Z–WTh, μm. Complementary graphs are shown in **S7 Fig**, **S1** and **S2** Videos files. Throughout the images, coordinates in some graphs were rotated at different angles to optimize display.

A 3D-modeling simulation was applied to address the limitations with 2D graphs. There were four steps to this method. For example, for the brain, IDs and WThs were calculated from the control linear regression equations (**S5 Table**) and placed in 3D-graphs (**Fig 14, S8 Fig**). Then, IDs and WThs for each of nineteen possible remodeling variants were calculated from the control equations and added to the 3D graphs. Every remodeling pattern was simulated by calculating its possible maximal deviation in dimensions that were arbitrarily limited to 300% for wall thickening or lumen widening, and 99% for wall thinning or lumen narrowing. IDs and WThs were then calculated from the hypertensive regression equations (**S5 Table**) and also added to 3D graphs. Finally, line proximity between real and predicted values was verified with methods of analytic geometry in 3D space[42]. From all possible variants the hypertensive remodeling in cerebral TAs corresponded only to the variant #9.

**Fig 15:**
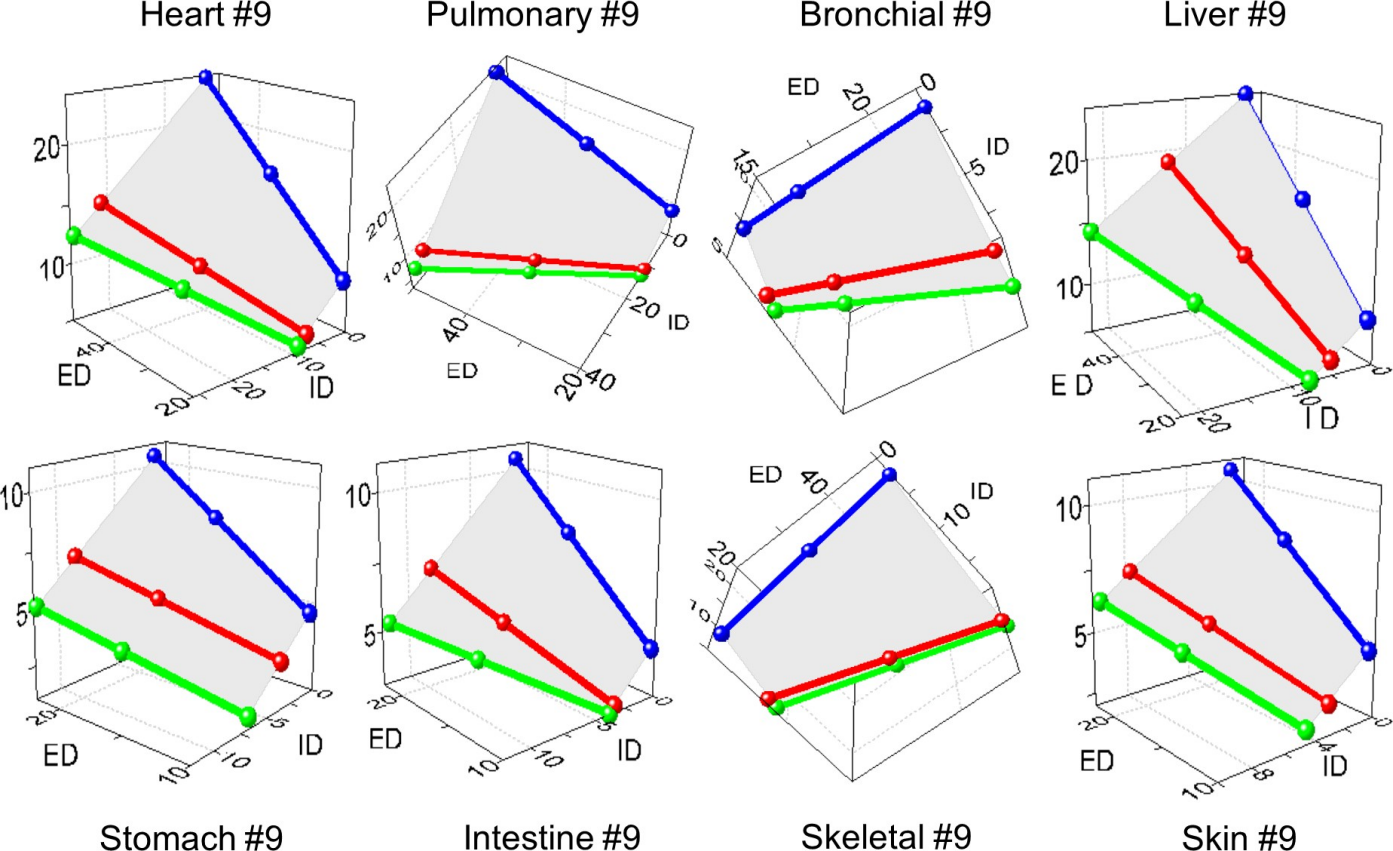
The common remodeling variant in hypertensive rats. The same variant #9 was found in most organs. Axis X–external diameter ED; axis Y–internal diameter (ID); axis Z–wall thickness (WTh), μm.

The brain, heart, lung, bronchi, liver, stomach, intestine, skin, and skeletal muscle developed the same remodeling variant #9, supporting the concept of uniform remodeling (**Fig 15**).

**Fig 16:**
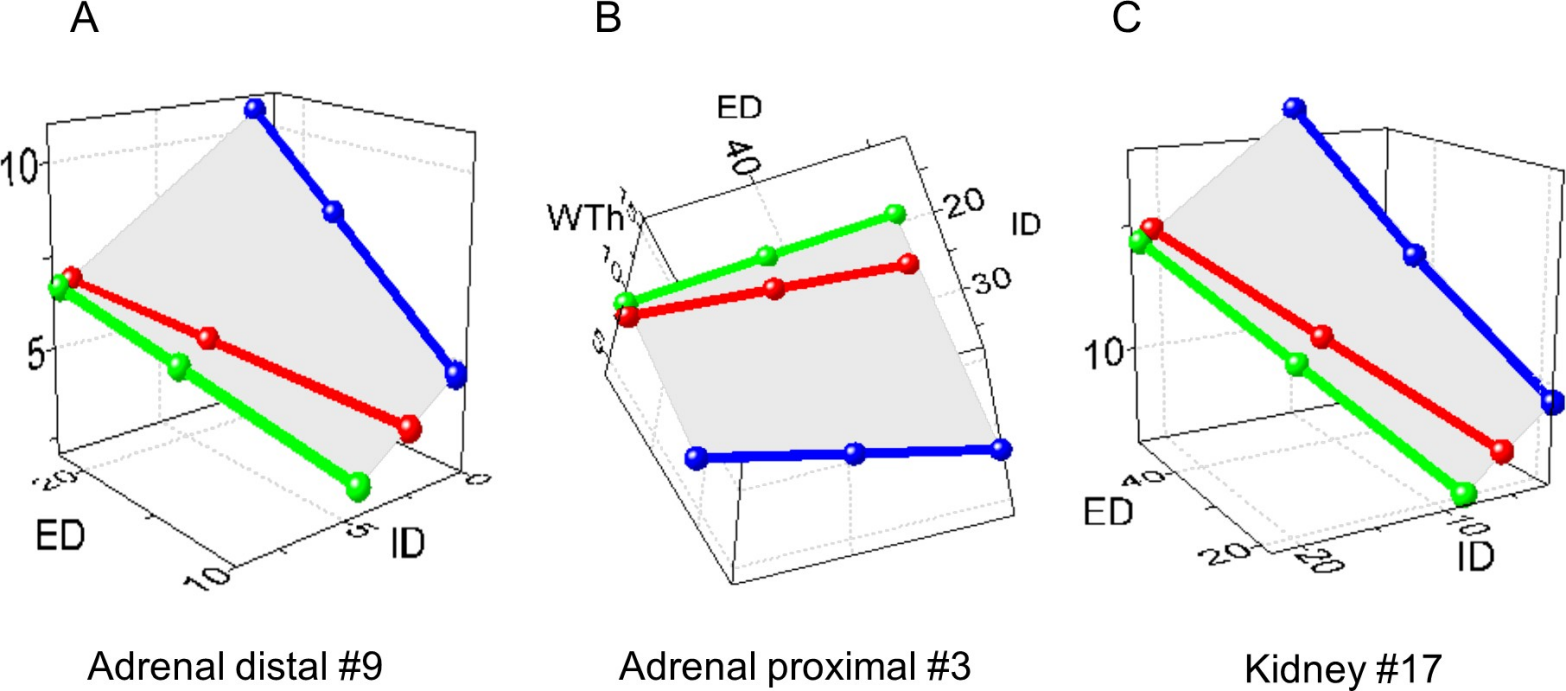
The distinct remodeling variants in hypertensive rats. (A) Adrenal distal segments of the external diameter (ED) ~10–20 μm were congruent with the variant #9, as other organs. (B) Adrenal proximal segments of ED ~30–50 μm were congruent with the variant #3. (C) The variant #17 appeared in the kidney. Axis X–external diameter ED; axis Y–internal diameter (ID); axis Z–wall thickness (WTh), μm.

The adrenal TAs exhibited more complex spatial rearrangements. The distal segments with ED ~10–20 μm followed the variant #9, as other organs (**Fig 16A).** The proximal segments of ED ≈ 30–50 μm transformed oppositely–through the variant #3 with a distended lumen and reduced WTh and MCSA (**Fig 16B)**. This was evident even on 2D graphs (**S6 Fig)**. Renal TAs developed the variant #17 (**Fig 16C**).

#### Connecting measurements to hemodynamic values

In addition to proposed TRR and TC, it was also desirable to estimate how media volume had changed in remodeling. The terminal media volume (TMV) could be calculated from complex profiles, similar to TC. However, in the current study we found that variation in accumulated frequencies would significantly affect TC and TMV values. The same negative issue for complex profiles to distinguish remodeling patterns was described above **(Fig 12A**). Accordingly, the linear regression equations, established for each organ, with counting ID and ED for the largest (50 μm) and smallest (10 μm) vessel calibers, provided more reliable estimates. TC and TMV were then computed by the formula for the truncated cone volume:

$$TC=\pi h*\left(IDlc^{2}+IDlc*\;IDsc+IDsc^{2}\right)/3$$ (5)

$$TMV=\pi h*\left(EDlc^{2}+EDlc*\;EDsc+EDsc^{2}\right)/3–TC$$ (6)

where EDlc, IDlc and EDsc, IDsc were the largest and smallest caliber limits, respectively, and *h* was the range of calibers (50 μm– 10 μm = 40 μm).

**Fig 17:**
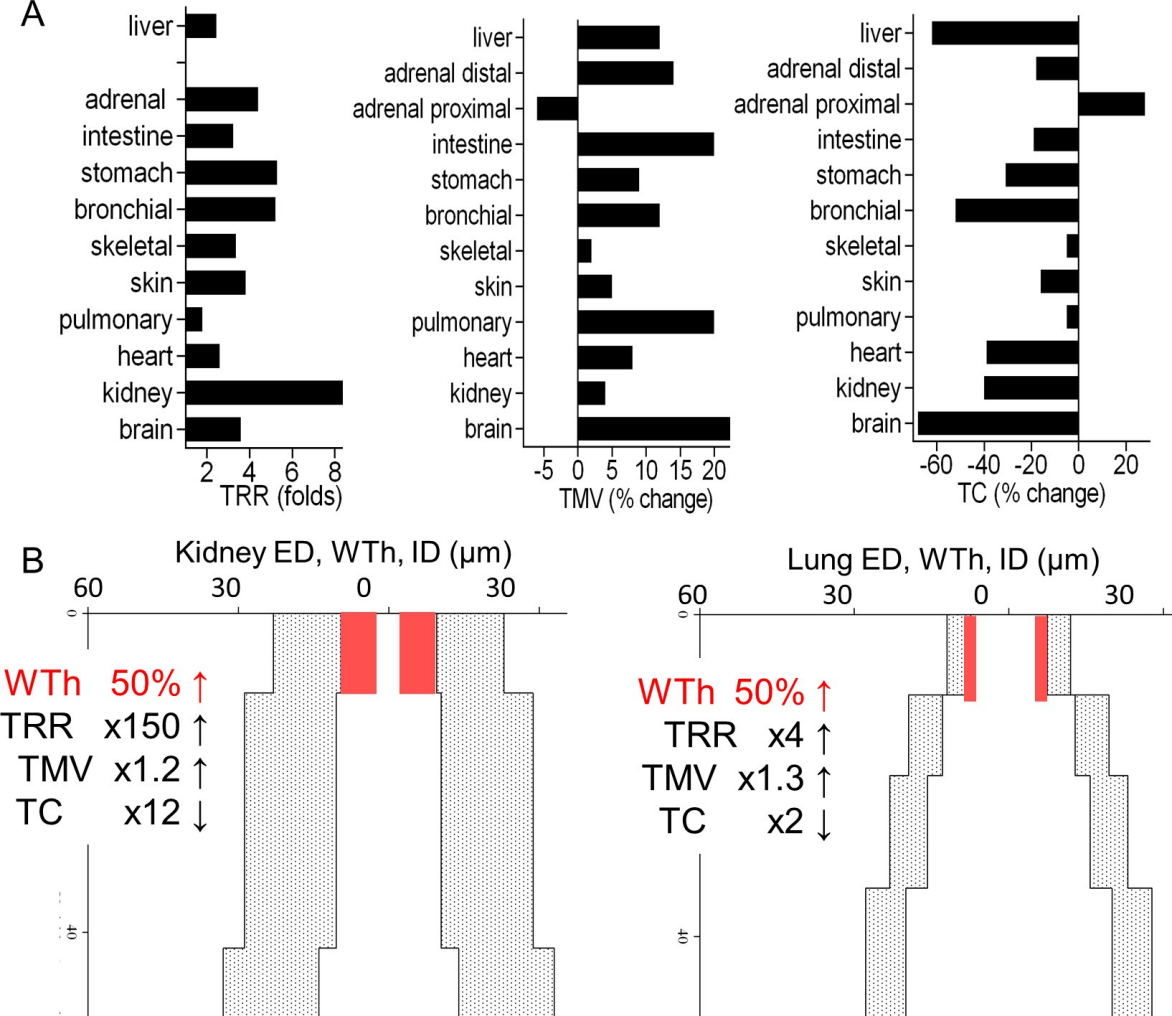
Hemodynamic parameters are enhanced variously in different organs. (A) There was no clear correlation between the values of terminal relative resistance (TRR), terminal capacity (TC), and terminal media volume (TMV). For example, in the brain enhanced TMV corresponded to a large drop in TC but a small increase in TRR. In the kidney a small elevation in TMV corresponded to a small lowering in TC but augmented TRR. (B) An equal 50% increase in wall expansion in kidney and lung arteries would cause marked differences in TRR and TC increases, while TMV would only increase mildly and remain comparable between the two organs. Different dynamics for TRR, TMV, and TC are the result of different initial organ specific dimensions. ED–external diameter; ID–internal diameter; WTh–wall thickness; x—fold increase.

Using this calculation, 1K1C hypertension induced a significant 2-5-fold increase in TRR in all organs (**Fig 17A**). It is noteworthy that the kidney has the highest increase in TRR (9-fold), while the pulmonary arteries–the lowest. The increased TRR and TC observed in the adrenal glands were due to different remodeling patterns in the proximal and distal arterial segments. In 1K1C rats TC demonstrated maximum decrease in the brain, liver and intestine, and minimal lowering in the skin, pulmonary and skeletal muscle arteries. TMV indicated arterial wall thickening, with maximum of 20–25% in the brain, intestine and pulmonary arteries, and minimum of 2–5% in skeletal muscle, skin and kidney, which were not proportional to TRR and TC. Organ-specific arterial dimensions could have significant impact on hemodynamic consequences of remodeling, contributing to the variable measures of TRR, TC, and TMV (**Fig 17B)**.

## Discussion

While the parenchyma of different organs is well represented on histological sections, arteries are an exception. They appear as circles and irregular strips with a wide range of sizes and shapes, which are difficult to quantify[30]. To date, there are two approaches to arterial morphometry. *In vitro* myography uses similar vascular segments to minimize variability. However, myography data are restricted to only a few areas that are suitable for sampling—mainly the mesentery and aorta. Second, quantification of intra-organ arteries is based on casual measurements of arterial segments on tissue sections. However, the majority of studies simply average the sizes and ratios, as for myography data, resulting in a high degree of variability and difficulty in comparing organs. Measured vessels are defined within uncertain ranges. For example, for the kidney: ‘arteries and arterioles’[43]; ‘intrarenal’[44],[45]; ‘proximal interlobular’[12]; ‘afferent arterioles’[46],[47]; ‘vessels adjacent to glomeruli in the outer cortex’[48]. Interlobular arteries could vary significantly in ID (~40 μm[12], ~25–50 μm[49], ~100 μm[50], ~30–250 μm[20]). The WLR is considered independent of diameters and not affected by the size of arteries studied[3],[9],[47], this matter has not been rigorously substantiated in the literature[4],[5],[29],[51]. Furthermore, we found as many as sixteen different formulae to calculate WLR. Some studies employed the diameter or radius: WTh/ID,[50],[52],[53]; ED—ID/2ID[54]; ED/ID[55]; WTh/IR[11][16]; a media index WTh/IR with IR specifically defined as the distance from the center of the arterial lumen to the middle point of the media[56]; 2WTh/ED[57],[58]; 2WTh/ID[59]; WTh/ED[60]; or ID/ED[61]. Others have measured WLR from the perimeters as EP/IP[46],[47],[48], or used the area: MCSA/LCSA[14],[15],[49]; MCSA/TCSA[44]; LCSA/MCSA[43]; LCSA/TCSA[62]. The WLR has also been interpreted as the ratio of the area to perimeter, MCSA/IP[20], or renamed as RI[63]. Rarely, point counting has been used to estimate WLR as the average ratio of the volume density of the walls to the volume density of the lumens[15],[64]. However, many studies indicate that WLR is not constant but declines 2-10-fold in arteries of ED 20–500 μm in the human and rat kidney[56],[65]; human and rat brain[66],[67]**; a**nd human and dog liver[34],[53]. Our data indicate that neither averaged WLR nor the mean values for ED, ID or WTh are applicable to even relatively small intervals of vessel caliber. Thus, any comparison of mean values for these measures is not appropriate[31].

To avoid averaging, some investigators also measured interval means. However, significant discrepancies in choice of interval steps and caliber ranges render such data as highly variable and difficult for comparative analysis[53],[64],[65],[66]. Thus the advantage of using intervals could be compromised by subjective choices of interval size that neither improves standardization nor comparison. We found the interval of 5 μm as optimal for all organs. That interval corresponds to adding one layer of vascular smooth muscle cells, which has a normal thickness of 5–7 μm[34],[68]. Indeed, the relation between WTh and the number of smooth muscle cell layers can be represented by a linear regression line (r = 0.88)[34].

Only a few attempts to apply linear regression to arterial morphometry have been published[17],[20], [34],[56]. Those studies demonstrated that the range of EDs from 10–100 μm fits linear regression, while wider intervals of ED 100–1000 μm follow an exponential function. We used the range of EDs from 10–50 μm as the most important to analyze. That caliber is not only the most frequently observed, but also best addresses the functional consequence of remodeling, being mainly responsible for regulation in peripheral resistance[3],[4],[5].

The hemodynamics of each organ are characterized by blood flow rate (BFR), regional vascular resistance (RVR), vascular volume, and percentage of cardiac output received[37],[38], reflecting the uniqueness of a vascular bed. Although each organ possesses a distinctive arterial tree, there are currently no standardized morphometric parameters to quantify those distinctions and correlate them to BFR or RVR[35],[36]. Indeed averaged EDs, IDs, and WLRs are only loosely associated with hemodynamic parameters, although they accompany impaired contraction or relaxation in *in vitro* myography experiments[67]. Our algorithm provides a promising solution to this problem. The use of linear regression equations allows for quantification of TRR, which reveals significant similarity to RVRs obtained by physiological methods (**Fig 6B and 6D**)[37],[38],[39]. The proposed TC formula also shows good correlation with BFR, determined by microsphere method[40] (**Fig 7B**). In rats renal TAs have thicker media and narrower lumens compared to mice, but lower resistance, which is consistent with hemodynamic data[69] (**Fig 8**).

There is a general assumption that in humans and animals, regardless of the type of hypertension, small arteries develop inward eutrophic or inward hypertrophic remodeling[7],[22]. Other studies have suggested that inward eutrophic remodeling occurs in essential hypertension, while secondary hypertension is associated with hypertrophic remodeling[4]. The concept of uniform remodeling is logical and reasonable since arteries throughout the body have similar structure and regulatory mechanisms. Nevertheless, studies using either random sections or *in vitro* myography present an entire spectrum of possible remodeling patterns. Differences in animal age, heterogeneous post-mortem arterial contraction, variable perfusion pressure, and different histoprocessing and morphometry techniques may account for data inconsistency[3],[70]. However, our attempt to find uniform remodeling in 1K1C rats by using the same tissue preparation and morphometry technique simultaneously in ten organs failed, and instead revealed distinctive, and even opposite patterns (**Table 2**). We also have found contradictions of two types in conventional remodeling analysis. First, statistical data from previous studies often cannot be assigned to a certain type of remodeling, since results do not fit the geometric formulae for annular ring dimensions. For example, mean ED and ID remains constant but WTh increased, or mean ID decreased while MCSA and WTh remain constant[28],[65],[71]. We applied the term “statistical artifact” (SA) to indicate them (**S4 Table)**. Second, results could be geometrically correct but showed different, opposite, or no remodeling patterns for the same model, organ, or arterial segments (**S3 and S4 Tables)**.

Many authors have presented data avoiding classification[13],[72]. Reviews on this subject have focused on the method of tissue preparation and the potential for sampling bias, and the general approach has not been revised[3],[22],[28],[51]. Indeed, the conventional classification was based on sketch-drawings, with assumptions regarding geometric parameters[10] (**Fig 9).** The classification presumed, for unclear reasons, the simultaneous movement of ED and ID as inward or outward. Such movement would occur if the artery remodeling is considered as simple inflation or deflation for an elastic, homogenous, gel-like structured thick-wall cylinder[73]. However, the arterial wall is categorized as a multilayered, helically arranged, fiber reinforced composite, with independent ED vs ID displacement[74]. Wall remodeling is a multilayered interaction involving hypertrophy, hyperplasia, apoptosis, hyalinosis and fibrinoid necrosis of vascular smooth muscle cells, as well as deposition of extracellular matrix [19],[75],[76],[77], that varies from inner to outer layers[72],[78]. We have found only one study that proposed a numerical approach to remodeling[28]. The numerical classification proposed here unambiguously defines any wall conformation, and could be applicable to remodeling not only in hypertension but diabetes, atherosclerosis[79], high or low blood flow[24],[53], restenosis[80], and bronchial remodeling[81].

Some studies considered that not WLR but other ratios such as EP/IP[47],[48], or MCSA/LCSA[12],[45],[49], or MCSA/IP[20] are more reliable and informative. Therefore we tested all possible dimensions and their ratios to demonstrate the lack of reliability in 2D graphs.

Calculation of RI is incorrect because it does not quantify any of the possible variants (**Fig 11**), and was designated to analyze only a remodeling pattern suggested by authors. GI does not bear any information about growth, as it simply counts the percentage change in MCSA that could be also assigned to many variants. This could explain variable RI and GI values found in in vitro myography experiments, which showed no connection between a remodeling pattern and RI and GI values (**S4 Table)**.

We also found two novel variants of remodeling, as predicted. The adrenals in the 1K1C model experienced a high blood flow. The blood, shunting from clipped or ligated main renal arteries, induced the variant #3 in the proximal segments, i.e. wall distention and thinning, as described for remodeling in high flow models[23],[24],[27]. However, distal TAs demonstrated the same variant #9 as in other organs. It may be that smallest arteries (arterioles) are structurally more resistant to distention from high blood flow, due to the absence of elastic laminae and adventitia[82]. Such segmental arterial remodeling has not been verified previously to our knowledge, and indeed was present in TAs with EDs ranging from 10–50 μm.

Reduced blood flow in stenotic kidneys[25],[26] should initiate one of the variants #11–14, which has been described for low-flow models[23],[27]. The renal arteries also experienced enhanced RAS activity that likely initiated the variant #9 in most organs. Development of the variant #17 might therefore reflect an interaction between flow-induced and pressure-induced stimuli. We speculated that remodeling by the variant #17 is a phenomenon of great importance that could be extended beyond the 1K1C model. It is well known that hypertension is combined with atherosclerosis in 70–80% of patients[83]. Therefore TAs in the brain and heart could be exposed to the low flow due to stenotic atherosclerotic plaques in proximal segments of cerebral and coronary arteries, and activated RAS as well. Presumably, TAs would develop the same variant #17 with the most increased resistance and reduced flow, which might be responsible for lesions in the heart and brain, being recognized as critical target organs.

We have provided the first quantitative evidence that pulmonary arteries respond to the hypertensive stimulus in 1K1C rats, and in the same remodeling pattern as other organs in the systemic circulation. Our data also represent the first evidence that substantial TAs remodeling occurs in the liver, intestine, and bronchi, which are not considered typical targets in hypertension[5],[51]. TRR correlated significantly with data obtained by physiological measures: in the Goldblatt model increased RVR has been shown in every organ, and is most pronounced in kidneys[84],[85],[86],[87]. In normal rats TC correlated with the regional BFR (**Fig 7**). However, it was difficult to compare our results with pathophysiological studies because the renal BFR in hypertensive animals has been shown as decreased, increased, or stable as well[84],[88],[89]. Evidently, hemodynamic correlations of TRR, TC and TMV need further investigation.

An important goal of our work was to determine if random tissue sections are a reliable source of data. Numerous studies of remodeling, exploring random tissue sections have been published in the past three decades, with most in the 1980s or 1990s, so the majority of our references are dated 15–20 years back. Since then *in vitro* myography studies dominate significantly, while morphometric studies on random sections have been infrequent.

For many years, post-mortem contraction has been considered a main cause of high variability in arterial morphometry[56]. Perfusion fixation was intended to eliminate post-mortem arterial contraction, especially with preliminary application of a vasodilator or vessel deactivation[28]. The present study indicates that perfusion fixation is not mandatory for morphometry on random tissue sections. In fact, perfused vs non-perfused organs or myograph experiments demonstrated similar stochastic remodeling patterns (**S3 and S4 Tables)**. Here, we avoided fixation deliberately, performing only immersion fixation in order to have animal (and potentially human) material in the same condition for comparison, since for the latter perfusion is unlikely to be applied. Our data demonstrate that careful consideration of arterial tapering is the most important factor for elaborate morphometry analysis. A similar approach has been widely accepted in atherosclerotic remodeling[79],[62].

In hypertension, progressive elevation of vascular resistance results from two components–functional (impaired contraction / relaxation) and structural (lumen narrowing from wall remodeling)[4],[5],[28]. The structural component can be recognized and quantified with histopathology analysis. However, the renal morphometry is dominated by glomerulosclerosis, tubular atrophy, and interstitial fibrosis, rather than angiopathy, long considered the hallmark of hypertension[77]. Studies in hypertensive models have demonstrated the critical necessity to quantify arterial remodeling as the structural component of vascular resistance. Current “conventional methods” utilise about 30 parameters depending on subjective choice: WLR that can be counted in 16 different ways; mean values for ED, ID, WTh; mean values for EP, IP; mean values for MCSA, LCSA; mean values for total cross sectional area and internal radius; any combination and ratios between parameters mentioned above; interval means of parameters mentioned above for different ranges depending on subjective preference; RI and GI. There is significant discrepancy with regards to the type of remodeling because the conventional methods are unable to verify the type of remodeling. For example, lumen narrowing can occur in any of nine possible variants (Fig 11). Each variant reflects different processes such as hypertrophy and/or hyperplasia of smooth muscle cells (#1, 9), atrophy and/or apoptosis and sclerosis (#11–14), cell rearrangement and sclerosis (#15–17)[76],[77]. The conventional methods are unable not only to quantify but even verify the type of remodeling, although this verification is of crucial importance. Despite the deficiencies in these models, they are still used to test treatment modalities. For example, ACE inhibitors demonstrate very significant prolonged positive effects in all animal models of hypertension, without exclusion[5],[7]. Nevertheless, the clinical data confirm that only 53% of patients with hypertension have their condition controlled to target levels[1]. Lumen narrowing in animal models is easily reversible while it progresses in humans. Transition between lumen dilation and stenosis is also poorly verified. Thus, a more refined analysis of arterial remodeling in different models, as proposed here, could help to solve this problem. In conclusion, we have developed an algorithm to quantify and standardize arterial remodeling analysis. Tissue sampling should be random and representative, according to known recommendations[30],[77]. Following fixation, sections must be stained with periodic acid–Schiff and Masson trichrome. Hematoxylin and eosin staining does not always clearly differentiate muscular and adventitial components, especially when hyalinosis, fibrinoid necrosis, or perivascular inflammatory infiltrates occur[77]. Random measurements of EDs and IDs are performed to obtain about 80–100 measurements of arteries with EDs between ~10–50 μm. Then, variables are arranged in order of increasing ED, divided into 5 μm intervals, and statistical analysis performed for each interval. Finally, the regression equations, complex profiles, remodeling variants, and hemodynamic parameters are computed from interval statistics and compared among models or organs. Once a remodeling variant is established, any phenotype markers, pathological lesions and dimensional conformations can be adequately compared along arterial trees in different organs **(Fig 10B).**The algorithm does not require additional counting or data gathering, compared to conventional morphometry of arteries on histological sections, and represents a more informative, standardized approach to morphometry of angiopathy.

## Supporting information

S1 Table Normality tests for averaged arterial dimensions in the kidney, heart and pulmonary arteries. (PDF)

S2 Table Linear regression equations from mean ± SD for 5 μm ED intervals. (PDF)

S3 Table Variable remodeling patterns in arteries studied on random histological sections. (PDF)

S4 Table Variable remodeling patterns in arteries studied via *in vitro* myography. (PDF)

S5 Table Linear regression equations of terminal arteries derived from mean ± SD for 5-μm ED intervals. All equations for hypertensive rats were significantly different from equations for control rats (*r*^2^ = 0.99; P < 0.0001).
(PDF)

S1 Fig Complex profiles revealed different tapering patterns in organs. Complementary graphs to **Fig 4A.** Axis X–the bidirectional common scale for the external diameter (ED, outer contours), internal diameter (ID, inner contours), wall thickness (WTh, shaded regions); axis Y–accumulated frequency of variables (%).
(PDF)

S2 Fig Linear regression equations and relative resistance in different organs. Lines and curves represent the best fit for different organs. Complementary graphs to **Fig 4B.** Points are mean ± SD for 5-μm ED intervals; *r^2^***—**goodness of fit coefficients. Corresponding equations are in **S2 Table.**
(PDF)

S3 Fig Comparison of linear regressions between different organs. Equations for lungs, bronchi, adrenal glands, stomach and skeletal muscles were very distinctive (P<0.0001). Brain and intestine (*) shared similar equations (P>0.41 for internal diameters (ID) and wall thickness (WTh)). Heart and spleen (**) were also close (P>0.43 for ID and >0.83 for WTh). Equations for kidney, liver and skin (***) were similar (P>0.77 for ID and >0.87 for WTh).
(PDF)

S4 Fig Histograms of terminal arteries in the kidney, heart and brain. The significant irregularity and asymmetry for dimensions in control and hypertensive rats. Data did not pass conventional statistical tests for normality (negative, P<0.001).
(PDF)

S5 Fig Control vs hypertensive complex profiles in organs. Control (green) and hypertensive (red) complex profiles were superimposed. Remodeling patterns are not recognizable. Complementary graphs to **Fig 12A.**
(PDF)

S6 Fig Linear regression lines displace similarly in most organs. The internal diameter slopes decreased (solid arrows), and the wall thickness slopes increased (dashed arrows). Adrenal arteries demonstrated opposite directions. Complementary graphs to **Fig 12B.**
(PDF)

S7 Fig The remodeling of renal arteries has been simulated for variants # 9–19. Displacement of linear regression lines up or down for any parameter was similar for many remodeling variants. Complementary graphs to **Fig 13.**
(PDF)

S8 Fig 3D-modeling simulation for the brain. No variants were congruent. Complementary graphs to **Fig 14.**
(PDF)

S1 Video 3-D modeling for the brain. The video demonstrates the variant #9 as congruent.
(AVI)

S2 Video 3-D modeling for the brain. The video demonstrates the variant #13 as an example of a non-congruent variant.
(AVI)
