# Supplementary material for: A novel method for comparison of arterial remodeling in hypertension: Quantification of arterial trees and recognition of remodeling patterns on histological sections
Source: PLoS One. 2019 May 21;14(5):e0216734. doi: 10.1371/journal.pone.0216734 (PMC6529011; doi:10.1371/journal.pone.0216734)
Supplement: S1 Table — (PDF) [file pone.0216734.s001.pdf]

**S1 Table.**

| Statistics | Kidney | | | Heart | | | Lung | | |
|---|---|---|---|---|---|---|---|---|---|
| | ED | ID | WTh | ED | ID | WTh | ED | ID | WTh |
| Mean ±SEM | 32.7±0.6 | 13.3±0.4 | 9.7± 0.2 | 26.6±1.0 | 12.7±0.6 | 6.9±0.3 | 29.2±1.3 | 20.9±1.0 | 4.1±0.2 |
| Minimum-Maximum | 17-50 | 7-27 | 5-16 | 13-50 | 3-34 | 2-16 | 8-76 | 4-60 | 2-10 |
| Kolmogorov-Smirnov normality test | P<0.001 No | P<0.001 No | P<0.001 No | P<0.001 No | P<0.001 No | P<0.001 No | P<0.0080 No | P<0.0260 No | P<0.0001 No |
| D'Agostino-Pearson normality test | P=0.0945 Yes | P=0.0642 Yes | P=0.1811 Yes | P<0.006 No | P<0.0001 No | P<0.0005 No | P=0.0007 No | P=0.0012 No | P=0.0010 No |
| Shapiro-Wilk normality test | P<0.0001 No | P<0.0001 No | P<0.0001 No | P<0.001 No | P<0.0001 No | P<0.0001 No | 0.0029 No | 0.0044 No | P<0.0001 No |

ED – external diameter; ID – internal diameter; WTh – wall thickness.
