# Supplementary material for: A novel method for comparison of arterial remodeling in hypertension: Quantification of arterial trees and recognition of remodeling patterns on histological sections
Source: PLoS One. 2019 May 21;14(5):e0216734. doi: 10.1371/journal.pone.0216734 (PMC6529011; doi:10.1371/journal.pone.0216734)
Supplement: S2 Table — (PDF) [file pone.0216734.s002.pdf]

S2 Table.

Brain	ID = 0.6966*ED - 3.526<br>WTh = 0.1517*ED + 1.785	$r^2 = 0.95$<br>$r^2 = 0.76$	P < 0.0001<br>P = 0.005
Kidney	ID = 0.4542*ED - 1.383<br>WTh = 0.2753*ED + 0.5603	$r^2 = 0.97$<br>$r^2 = 0.99$	P = 0.0001<br>P = 0.0001
Heart	ID = 0.5486*ED - 2.020<br>WTh = 0.2257*ED + 1.010	$r^2 = 0.98$<br>$r^2 = 0.97$	P < 0.0001<br>P < 0.0001
Pulmonary arteries	ID = 0.8099*ED - 2.697<br>WTh =0.098*ED + 1.221	$r^2 = 0.99$<br>$r^2 = 0.95$	P < 0.0001<br>P < 0.0001
Skin	ID = 0.4049*ED + 0.6122<br>WTh = 0.2996*ED - 0.4020	$r^2 = 0.97$<br>$r^2 = 0.98$	P < 0.0001<br>P < 0.0001
Skeletal muscle	ID = 0.3767*ED + 1.198<br>WTh = 0.3348*ED - 0.7042	$r^2 = 0.99$<br>$r^2 = 0.95$	P < 0.0001<br>P = 0.0009
Bronchial arteries	ID = 0.7064*ED – 7.196<br>WTh = 0.1659*ED + 3.050	$r^2 = 0.93$<br>$r^2 = 0.77$	P = 0.0004<br>P = 0.0097
Stomach	ID = 0.4113*ED + 2.723<br>WTh = 0.2952*ED - 1.395	$r^2 = 0.89$<br>$r^2 = 0.94$	P =0.0005<br>P < 0.0001
Intestine	ID = 0.6336*ED - 2.494<br>WTh = 0.1840*ED + 1.194	$r^2 = 0.98$<br>$r^2 = 0.96$	P < 0.0001<br>P < 0.0001
Adrenal	ID = 0.3535*ED + 0.948<br>WTh = 0.3232*ED - 0.4742	$r^2 = 0.96$<br>$r^2 = 0.99$	P < 0.0001<br>P < 0.0001
Liver	ID = 0.4652*ED - 1.533<br>WTh= 0.2719*ED + 0.6479	$r^2 = 0.90$<br>$r^2 = 0.92$	P = 0.0003<br>P = 0.0002
Spleen	ID = 0.5398*ED – 1.417<br>WTh = 0.2301*ED + 0.7083	$r^2 = 0.99$<br>$r^2 = 0.99$	P < 0.0001<br>P < 0.0001

ED – external diameter; ID – internal diameter; WTh – wall thickness.
