# Supplementary material for: A novel method for comparison of arterial remodeling in hypertension: Quantification of arterial trees and recognition of remodeling patterns on histological sections
Source: PLoS One. 2019 May 21;14(5):e0216734. doi: 10.1371/journal.pone.0216734 (PMC6529011; doi:10.1371/journal.pone.0216734)
Supplement: S3 Table — (PDF) [file pone.0216734.s003.pdf]

S3 Table. Variable remodeling patterns in arteries studied on random histological sections.

Author	Experimental model	Location of arteries	NC
**Liu et al.**[1]	2K1C (PF)	renal arterioles 30-60µm	**9**
		> 60µm	**--**
		renal arterioles 30-60µm	**9**
		> 60µm	**--**
**Helmchen et al.**[2]	2K1C (IF)	interlobular	**2**
**Qin et al.**[3]	2K1C(PF)	mesentery	**1**
**Korsgaard et al.**[4]	1K1C, unfixed, wire myograph	mesenteric III branch	**17**
		renal arcuate arteries	**--**
**Deng et al.**[5]	wire myograph, 2K1C	mesentery III order	**17**
	1K1C	mesentery III order	**16**
**Zhou et al.**[6]	2K1C, wire myograph	renal arteries	**17**
**Kinuno et al.**[7]	SHR (PF)	renal interlobular	**2**
	SHR+uninephrectomy (PF)	renal interlobular	**4**
**Owens et al.**[8]	SHR (PF)	mesentery, I branch	**16**
		II branch	**--**
		III - IV branch	**17**
**Smeda et al.**[9]	SHR (PF)	renal, ID~20-60µm	**--**
		renal, ID~300-60µm	**2**
**Johansson B.**[10]	SHR (PF)	cerebral extracranial	**6**
		cerebral intracranial	**2**
	SHRSP (PF)	cerebral extracranial	**8**
		cerebral intracranial	**4**
**Nordborg et al.**[11]	SHR (IF)	IF, mesentery	**17**
	SHRSP (IF)	mesentery	**16**
	SHR (IF)	renal	**2**
	SHRSP (IF)	renal	**6**
**Leh et al.**[12]	SHR (IF)	renal afferent arterioles	**6**
**Ohara et al.**[13]	SHR (PF)	PF, renal interlobular	**9**
**Kost et al.**[14]	SHR (PF)	renal afferent	**--**
		interlobular	**--**
		arcuate	**18**
	SHR+AngII (PF)	renal afferent	**--**
		interlobular	**2**
		arcuate	**2**
**Lee et al.**[15]	SHR (PF)	mesentery, ID=120-250 µm	**2**
**Limas et al.**[16]	SHR, age dependent (PF)	renal, 10 weeks	**--**
		ED= 50-100 µm 20 weeks	**2**
		28 weeks	**14**
		48 weeks	**9**
**Casare et al.**[17]	AngII (PF)	renal afferent interlobular	**7**
			**4**
**Mazzali et al.**[18]	Hyperuricemia (IF)	renal afferent arterioles	**1**

2K1C, two kidney-one clip; Ang, angiotensin; DOCA, deoxycorticosterone acetate; IF, immersion fixed; PF, perfusion fixed; SHR, spontaneously hypertensive rats; SHRSP, spontaneously hypertensive rats stroke prone; NC, numerical classification.

References for S3 Table

1. Liu JL, Bishop SP, Overbeck HW. Morphometric evidence for non-pressure-related arterial wall thickening in hypertension. Circ Res. 1988 May;62(5):1001–10.
2. Helmchen U, Kneissler U, Bohle RM, Reher A, Groene HJ. Adaptation and decompensation of intrarenal small arteries in experimental hypertension. J Cardiovasc Pharmacol. 1984;6:S696-705.
3. Qin X-P, Zeng S-Y, Tian H-H, Deng S-X, Ren J-F, Zheng Y-B, et al. Involvement of prolylcarboxypeptidase in the effect of rutaecarpine on the regression of mesenteric artery hypertrophy in renovascular hypertensive rats. Clin Exp Pharmacol Physiol. 2009 Mar;36(3):319–24.
4. Korsgaard N, Mulvany MJ. Cellular hypertrophy in mesenteric resistance vessels from renal hypertensive rats. Hypertens (Dallas, Tex 1979). 1988 Aug;12(2):162–7.
5. Deng LY, Schiffrin EL. Morphological and functional alterations of mesenteric small resistance arteries in early renal hypertension in rats. Am J Physiol. 1991 Oct;261(4 Pt 2):H1171-7.
6. Zhou N, Wang T, Song J, He H, He J, He L. Antihypertensive and vascular remodelling effects of the imperatorin derivative OW1 in renovascular hypertension rats. Clin Exp Pharmacol Physiol. 2014 Aug;41(8):571–8.
7. Kinuno H, Tomoda F, Koike T, Takata M, Inoue H. Effects of uninephrectomy on renal structural properties in spontaneously hypertensive rats. Clin Exp Pharmacol Physiol. 2005 Mar;32(3):173–8.
8. Owens GK, Schwartz SM, McCanna M. Evaluation of medial hypertrophy in resistance vessels of spontaneously hypertensive rats. Hypertension. 1988 Feb;11(2):198–207.
9. Smeda JS, Lee RM, Forrest JB. Structural and reactivity alterations of the renal vasculature of spontaneously hypertensive rats prior to and during established hypertension. Circ Res. 1988 Sep;63(3):518–33.
10. Johansson BB. Cerebral vascular bed in hypertension and consequences for the brain. Hypertension. 1984;6(6 Pt 2):III81-6.
11. Nordborg C, Ivarsson H, Johansson BB, Stage L. Morphometric study of mesenteric and renal arteries in spontaneously hypertensive rats. J Hypertens. 1983 Dec;1(4):333–8.
12. Leh S, Hultström M, Rosenberger C, Iversen BM. Afferent arteriolopathy and glomerular collapse but not segmental sclerosis induce tubular atrophy in old spontaneously hypertensive rats. Virchows Arch. 2011 Jul;459(1):99–108.
13. Ohara M, Tomoda F, Koike T, Liu H, Uno K, Nitta A, et al. Pubertal administration of antiserum against nerve growth factor regresses renal vascular remodeling in spontaneously hypertensive rats. Clin Exp Pharmacol Physiol. 2015 Jun;42(6):687–94.
14. Kost CK, Herzer WA, Li P, Notoya M, Mizuhira V, Inagami T, et al. Angiotensin II-induced structural and functional alterations in spontaneously hypertensive rat kidney. Am J Physiol. 1996 Jan;270(1 Pt 2):F229-36.
15. Lee RM, Smeda JS. Primary versus secondary structural changes of the blood vessels in hypertension. Can J Physiol Pharmacol. 1985 Apr;63(4):392–401.
16. Limas C, Westrum B, Limas CJ. The evolution of vascular changes in the spontaneously hypertensive rat. Am J Pathol. 1980 Feb;98(2):357–84.
17. Casare FAM, Thieme K, Costa-Pessoa JM, Rossoni LV, Couto GK, Fernandes FB, et al. Renovascular remodeling and renal injury after extended angiotensin II infusion. Am J Physiol - Ren Physiol. 2016 Jun 1;310(11):F1295–307.
18. Mazzali M, Kanellis J, Han L, Feng L, Xia YY, Chen Q, et al. Hyperuricemia induces a primary renal arteriolopathy in rats by a blood pressure-independent mechanism. Am J Physiol Ren Physiol. 2002;282(6):F991-7.
