# Supplementary material for: A novel method for comparison of arterial remodeling in hypertension: Quantification of arterial trees and recognition of remodeling patterns on histological sections
Source: PLoS One. 2019 May 21;14(5):e0216734. doi: 10.1371/journal.pone.0216734 (PMC6529011; doi:10.1371/journal.pone.0216734)
Supplement: S4 Table — (PDF) [file pone.0216734.s004.pdf]

S4 Table Variable remodeling patterns in arteries studied via *in vitro* myography.

Author	Experimental model	Arterial location	NC	RI	GI
**Baumbach et al.**[19]	1K1C (PM)	deactivated cerebral	**SA**	-220	26
**Korsgaard et al.**[4]	1K1C (WM)	mesenteric III branch	**17**	0.7	0.4
		renal arcuate	-	0.7	0.1
**Deng et al.**[5]	2K1C (WM)	mesentery III order	**16**	0.9	0.1
	1K1C (WM)	mesentery III order	**17**	0.8	0.2
**Zhou et al.**[6]	2K1C (WM)	renal arteries	**17**	8	0.2
**Mulvany et al.**[20]	SHR (WM)	mesenteric	**2**	75	26
**Bund et al.**[21]	SHR (PM)	mesenteric	**2**	18	56
		femoral IV branch	**SA**	103	-5
**Briones et al.**[22]	SHR(PM)	mesenteric	**16**	98	0.3
**Baumbach et al.**[19]	SHR (PM)	deactivated cerebral	**17**	-1	58
**Baumbach et al.**[23]	SHRSP (PM)	basilar baseline	**9**	34	40
		basilar deactivated	**17**	76	24
**New et al.**[24]	SHR (PM)	cremaster	**SA**	62	28
		cerebral	**2**	-3	48
		mesenteric	**16**	84	23
**Dao et al.**[25]	AngII (PM)	mesenteric	**SA**	-21	32
		mesenteric	**2**	316	54
**De Ciuceis et al.**[26]	AngII (PM)	mesenteric	**2**	52	20
	AngII in op/+ mice (PM)	mesenteric	**16**	106	-5
**Neves et al.**[27]	AngII (PM)	mesenteric	**2**	67	31
	Aldosteron (PM)	mesenteric	**SA**	64	30
**Briones et al.**[28]	ouabain (PM)	mesenteric	**15**	137	-15
**Baumbach et al.**[29]	L-NAME (PM)	cerebral	**SA**	111	32
	eNOS -/- (PM)		**5**		
**Baumbach et al.**[30]	CuZn SOD deficient (PM)	cerebral	**SA**	458	86
**Rizzoni et al.**[31]	Human, EHT (WM)	gluteal biopsy,	**16**	104	6
**Rizzoni et al.**[32]	Human, EHT (WM)	gluteal biopsy	**17**	86	14
	RVH (WM)		**15**	108	-14
**Rizzoni et al.**[33]	Human, EHT (WM)	gluteal biopsy	**2**	86	17
	RVH (WM)		**17**	72	48
**Endemann et al.**[34]	Human, , EHT (WM)	gluteal biopsy	**SA**	110	18
	DM2+EHT (PM)		**2**	45	126

1K1C, one kidney-one clip; 2K1C, two kidney-one clip; Ang, angiotensin; DM2, diabetes mellitus type 2; EHT, essential hypertension; eNOS, endothelial nitric oxide synthase; GI, growth index; L-NAME, Nitro-L-arginine methyl ester; op/+, osteopetrotic heterozygous; NC, numerical classification; PM, pressure myograph; RI, remodeling index; RVH, renovascular hypertension; SA, statistical artifact; SHR, spontaneously hypertensive rats; SHRSP, spontaneously hypertensive rats stroke prone; SOD, superoxide dismutase; WM, wire myograph.

References for S4 Table

19. Baumbach GL, Hajdu MA. Mechanics and composition of cerebral arterioles in renal and spontaneously hypertensive rats. Hypertens (Dallas, Tex 1979). 1993 Jun;21(6 Pt 1):816–26.
20. Mulvany MJ, Baandrup U, Gundersen HJ. Evidence for hyperplasia in mesenteric resistance vessels of spontaneously hypertensive rats using a three-dimensional disector. Circ Res. 1985 Nov;57(5):794–800.
21. Bund SJ, Lee RMKW. Arterial structural changes in hypertension: a consideration of methodology, terminology and functional consequence. J Vasc Res. 2003;40(6):547–57.
22. Briones AM, González JM, Somoza B, Giraldo J, Daly CJ, Vila E, et al. Role of elastin in spontaneously hypertensive rat small mesenteric artery remodelling. J Physiol. 2003 Oct 1;552(Pt 1):185–95.
23. Baumbach GL, Heistad DD. Remodeling of cerebral arterioles in chronic hypertension. Hypertension. 1989 Jun;13(6 Pt 2):968–72.
24. New DI, Chesser AM, Thuraisingham RC, Yaqoob MM. Structural remodeling of resistance arteries in uremic hypertension. In: Kidney International. 2004. p. 1818–25.
25. Dao HH, Martens FM, Larivière R, Yamaguchi N, Cernacek P, de Champlain J, et al. Transient involvement of endothelin in hypertrophic remodeling of small arteries. J Hypertens. 2001 Oct;19(10):1801–12.
26. De Ciuceis C, Amiri F, Brassard P, Endemann DH, Touyz RM, Schiffrin EL. Reduced vascular remodeling, endothelial dysfunction, and oxidative stress in resistance arteries of angiotensin II-infused macrophage colony-stimulating factor-deficient mice: evidence for a role in inflammation in angiotensin-induced vascular injury. Arterioscler Thromb Vasc Biol. 2005 Oct 1;25(10):2106–13.
27. Neves MF, Virdis A, Schiffrin EL. Resistance artery mechanics and composition in angiotensin II-infused rats: effects of aldosterone antagonism. J Hypertens. 2003 Jan;21(1):189–98.
28. Briones AM, Xavier FE, Arribas SM, González MC, Rossoni L V, Alonso MJ, et al. Alterations in structure and mechanics of resistance arteries from ouabain-induced hypertensive rats. Am J Physiol Heart Circ Physiol. 2006 Jul 24;291(1):H193-201.
29. Baumbach GL, Sigmund CD, Faraci FM. Structure of cerebral arterioles in mice deficient in expression of the gene for endothelial nitric oxide synthase. Circ Res. 2004 Oct 15;95(8):822–9.
30. Baumbach GL, Didion SP, Faraci FM. Hypertrophy of Cerebral Arterioles in Mice Deficient in Expression of the Gene for CuZn Superoxide Dismutase. Stroke. 2006 Jul 1;37(7):1850–5.
31. Rizzoni D, De Ciuceis C, Porteri E, Paiardi S, Boari GE, Mortini P, et al. Altered structure of small cerebral arteries in patients with essential hypertension. J Hypertens. 2009 Apr;27(4):838–45.
32. Rizzoni D, Porteri E, De Ciuceis C, Boari GEM, Zani F, Miclini M, et al. Lack of prognostic role of endothelial dysfunction in subcutaneous small resistance arteries of hypertensive patients. J Hypertens. 2006 May;24(5):867–73.
33. Rizzoni D, Porteri E, Castellano M, Bettoni G, Muiesan ML, Tiberio G, et al. Endothelial dysfunction in hypertension is independent from the etiology and from vascular structure. Hypertens (Dallas, Tex 1979). 1998 Jan;31(1 Pt 2):335–41.
34. Endemann DH, Pu Q, De Ciuceis C, Savoia C, Virdis A, Neves MF, et al. Persistent remodeling of resistance arteries in type 2 diabetic patients on antihypertensive treatment. Hypertens (Dallas, Tex 1979). 2004 Feb;43(2):399–404.
