# Supplementary material for: A novel method for comparison of arterial remodeling in hypertension: Quantification of arterial trees and recognition of remodeling patterns on histological sections
Source: PLoS One. 2019 May 21;14(5):e0216734. doi: 10.1371/journal.pone.0216734 (PMC6529011; doi:10.1371/journal.pone.0216734)
Supplement: S5 Table — All equations for hypertensive rats were significantly different from equations for control rats (r2 = 0.99; P < 0.0001). (PDF) [file pone.0216734.s005.pdf]

S5 Table.

Organ	Control vessels	1K1C hypertension
**Brain**	ID= 0.6966*ED - 3.526<br>WTh= 0.1517*ED + 1.785	ID= 0.4470*ED - 1.429<br>WTh= 0.2674*ED + 0.9145
**Kidney**	ID= 0.4542*ED - 1.383<br>WTh= 0.2753*ED + 0.5603	ID= 0.5185*ED - 5.476<br>WTh= 0.2437*ED + 2.573
**Heart**	ID= 0.5486*ED - 2.020<br>WTh= 0.2257*ED + 1.010	ID= 0.4485*ED - 1.415<br>WTh= 0.2758*ED + 0.7073
**Pulmonary arteries**	ID= 0.8229*ED - 3.187<br>WTh= 0.0945*ED +1.356	ID= 0.75160*ED - 3.183<br>WTh= 0.1242*ED + 1.592
**Skin**	ID= 0.4049*ED + 0.6122<br>WTh= 0.2996*ED - 0.4020	ID= 0.3736*ED - 0.5334<br>WTh= 0.3180*ED + 0.0852
**Skeletal muscle**	ID= 0.3670*ED + 1.280<br>WTh= 0.3363*ED - 0.8806	ID= 0.3724*ED + 0.0860<br>WTh= 0.3207*ED - 0.3888
**Bronchial arteries**	ID= 0.7064*ED – 7.196<br>WTh= 0.1669*ED + 3.050	ID= 0.5833*ED – 6.041<br>WTh = 0.2261*ED + 2.33
**Stomach**	ID= 0.4113*ED + 2.723<br>WTh= 0.2952*ED - 1.395	ID= 0.4039*ED – 0.3570<br>WTh= 0.2981*ED + 0.1785
**Intestine**	ID= 0.6336*ED - 2.494<br>WTh=0.1840*ED + 1.194	ID= 0.3978*ED - 0.5241<br>WTh = 0.3011*ED + 0.2620
**Adrenal**	ID= 0.3535*ED + 0.9484<br>WTh= 0.3232*ED - 0.4742	ID= 0.4908*ED - 2.486<br>WTh= 0.2482*ED +1.296
**Liver**	ID= 0.4652*ED - 1.533<br>WTh= 0.2719*ED + 0.6479	ID= 0.2322*ED + 0.6379<br>WTh= 0.3917*ED - 0.6073

ED – external diameter; ID – internal diameter; WTh – wall thickness.
