# Supplementary figures and images for: A novel method for comparison of arterial remodeling in hypertension: Quantification of arterial trees and recognition of remodeling patterns on histological sections
Source: PLoS One. 2019 May 21;14(5):e0216734. doi: 10.1371/journal.pone.0216734 (PMC6529011; doi:10.1371/journal.pone.0216734)

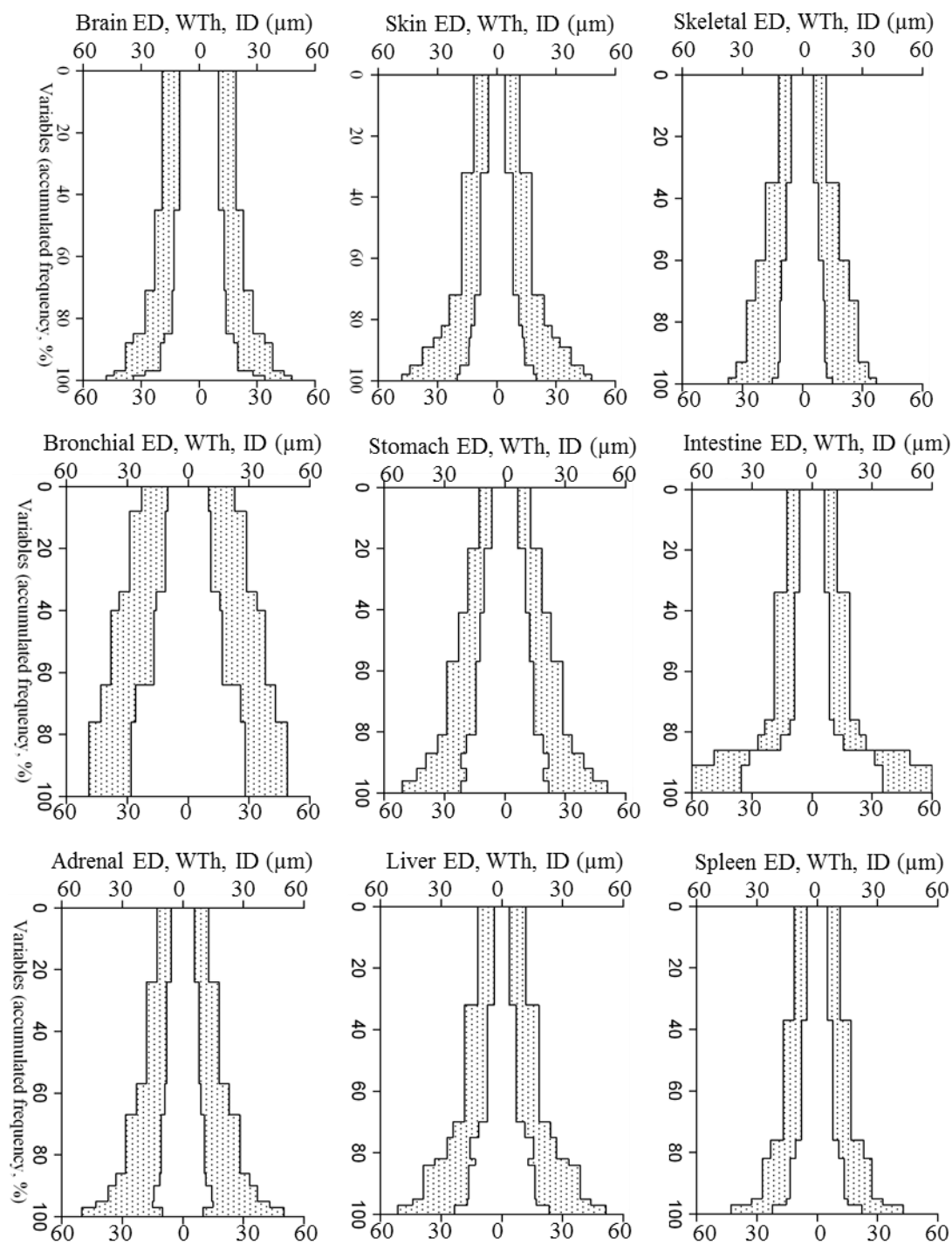

ED – external diameter; ID – internal diameter; WTh – wall thickness.

**S1 Fig.**

Supplement: S1 Fig — Complementary graphs to Fig 4A. Axis X–the bidirectional common scale for the external diameter (ED, outer contours), internal diameter (ID, inner contours), wall thickness (WTh, shaded regions); axis Y–accumulated frequency of variables (%). (PDF) [file pone.0216734.s006.pdf]

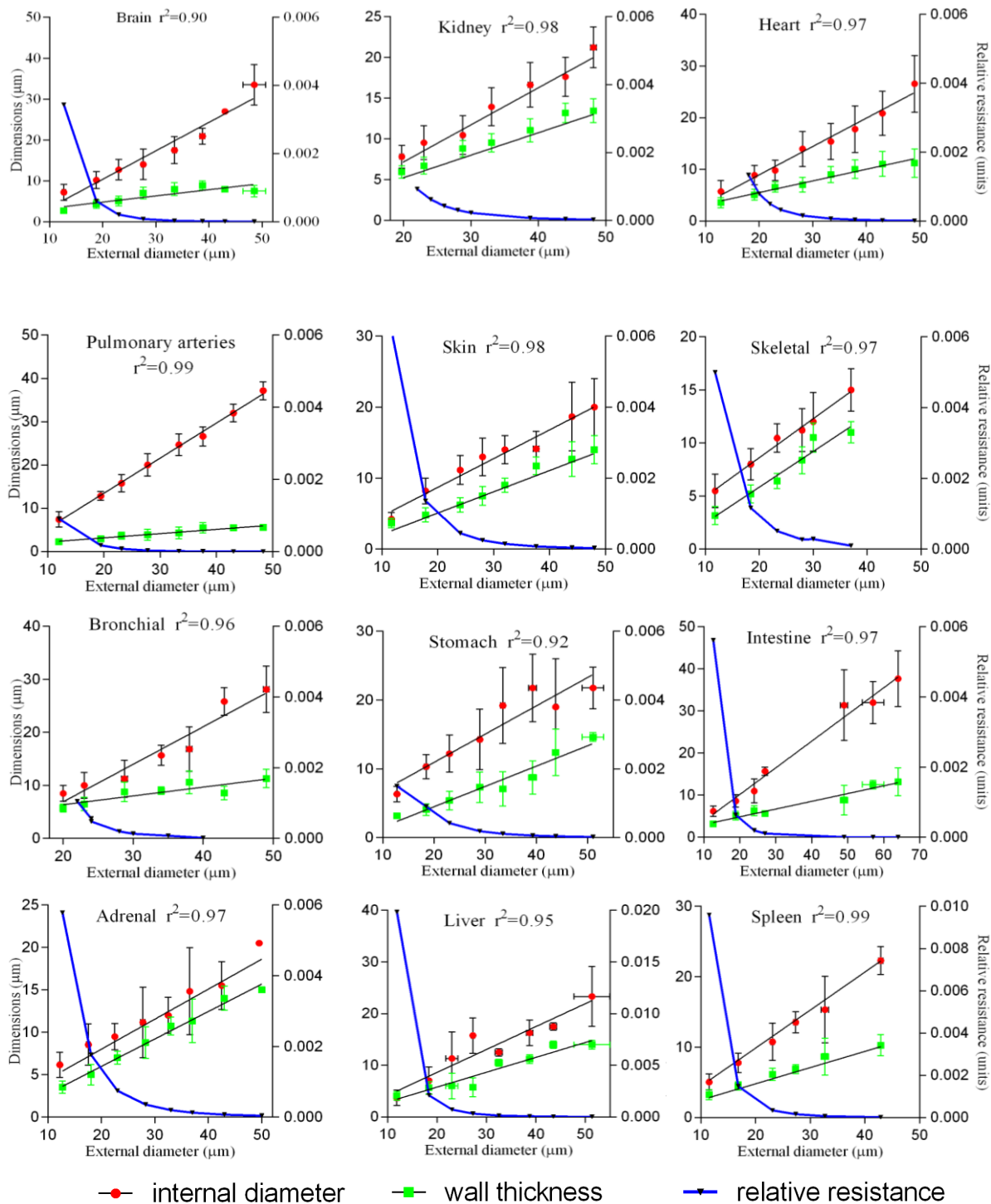

S2 Fig.

Supplement: S2 Fig — Lines and curves represent the best fit for different organs. Complementary graphs to Fig 4B. Points are mean ± SD for 5-μm ED intervals; r2—goodness of fit coefficients. Corresponding equations are in S2 Table. (PDF) [file pone.0216734.s007.pdf]

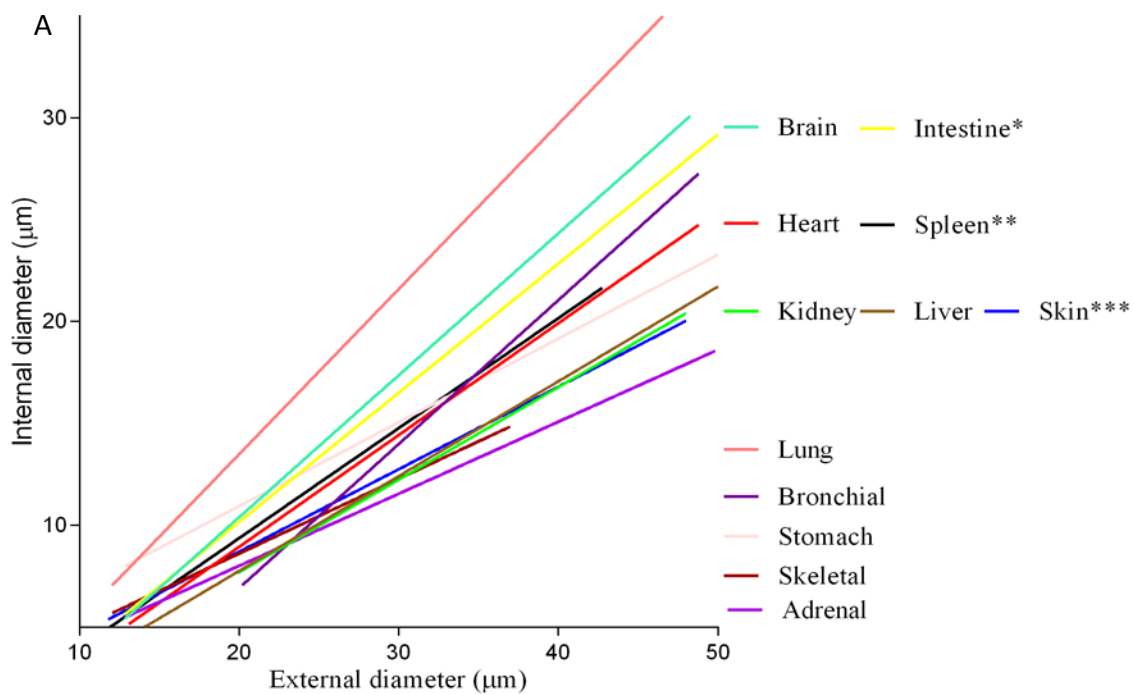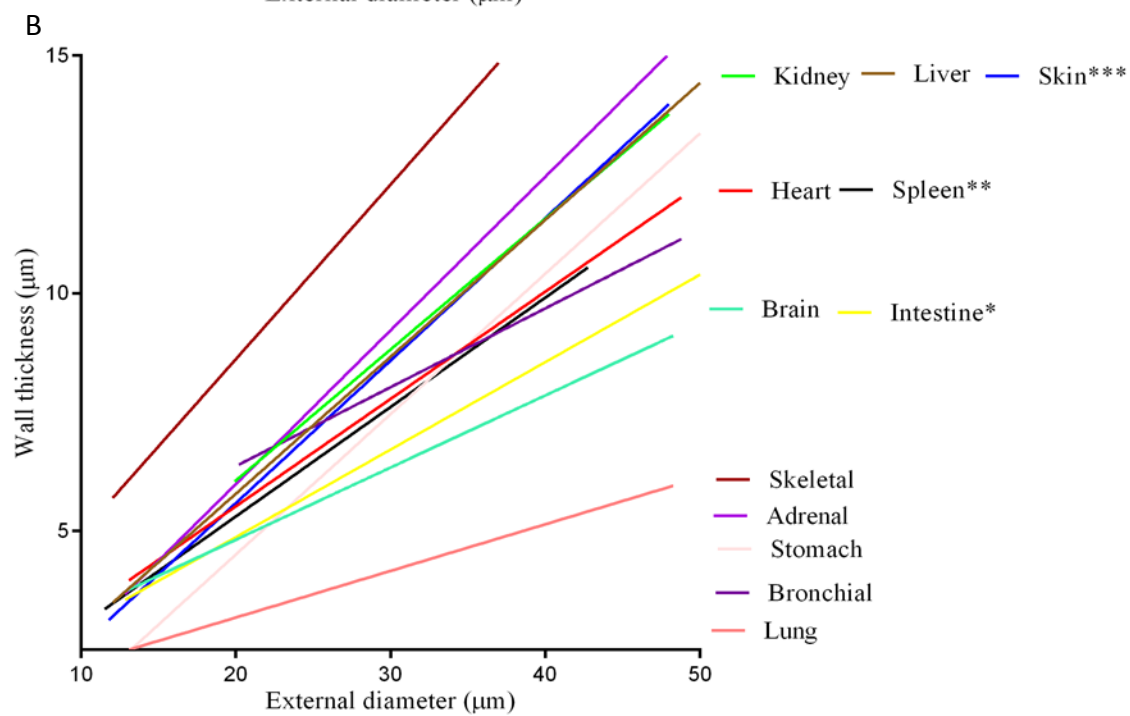

**S3 Fig.**

Supplement: S3 Fig — Equations for lungs, bronchi, adrenal glands, stomach and skeletal muscles were very distinctive (P<0.0001). Brain and intestine (*) shared similar equations (P>0.41 for internal diameters (ID) and wall thickness (WTh)). Heart and spleen (**) were also close (P>0.43 for ID and >0.83 for WTh). Equations for kidney, liver and skin (***) were similar (P>0.77 for ID and >0.87 for WTh). (PDF) [file pone.0216734.s008.pdf]

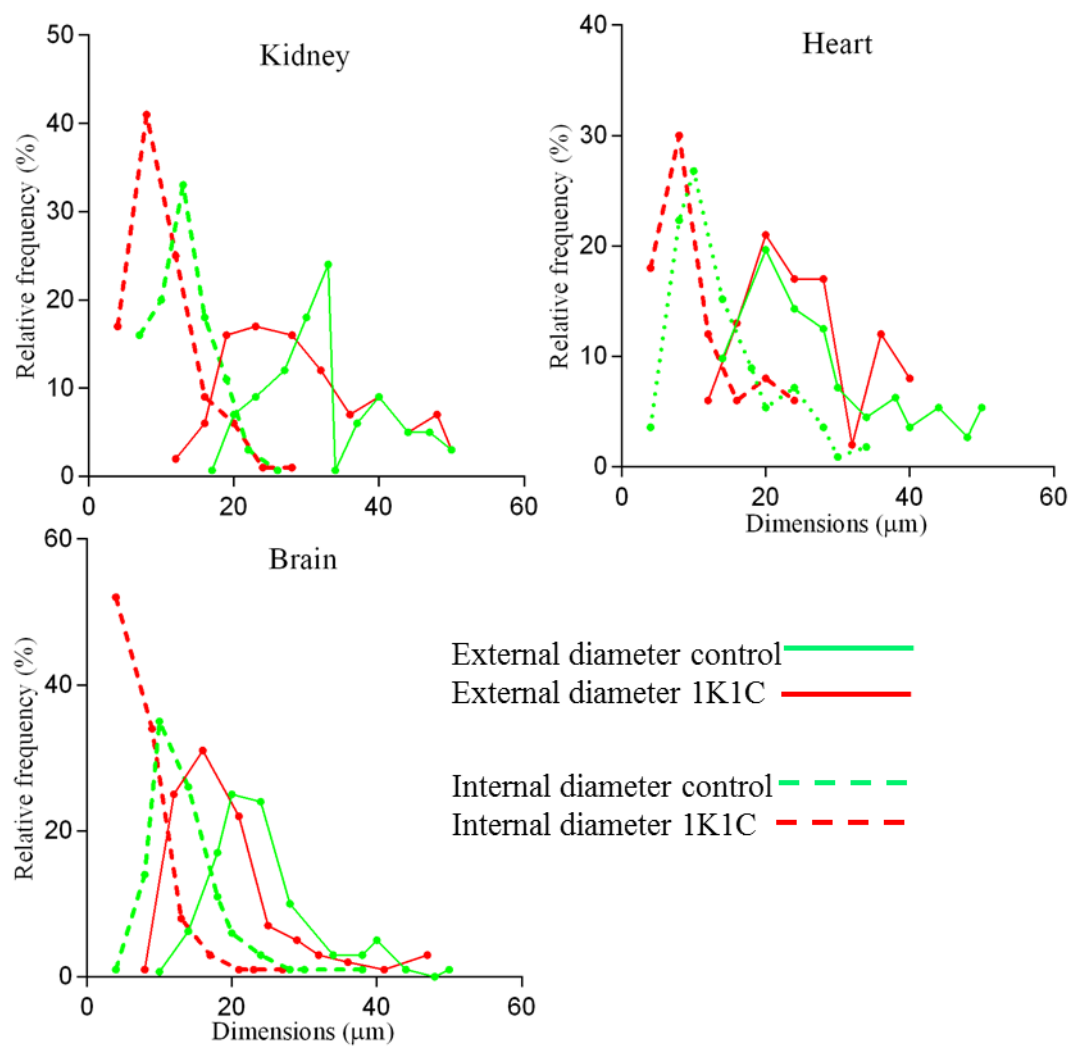

**S4 Fig.**

Supplement: S4 Fig — The significant irregularity and asymmetry for dimensions in control and hypertensive rats. Data did not pass conventional statistical tests for normality (negative, P<0.001). (PDF) [file pone.0216734.s009.pdf]

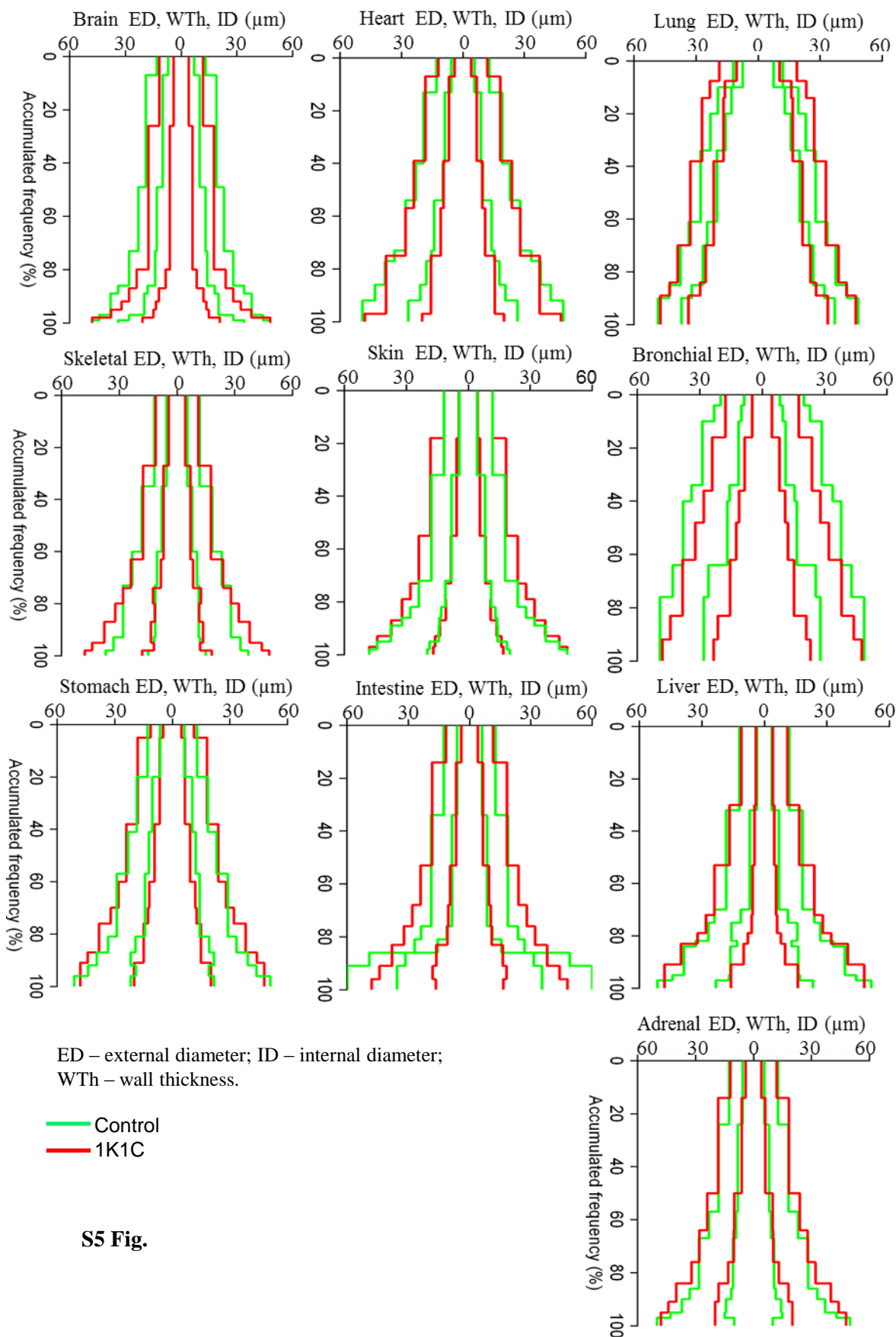

Supplement: S5 Fig — Control (green) and hypertensive (red) complex profiles were superimposed. Remodeling patterns are not recognizable. Complementary graphs to Fig 12A. (PDF) [file pone.0216734.s010.pdf]

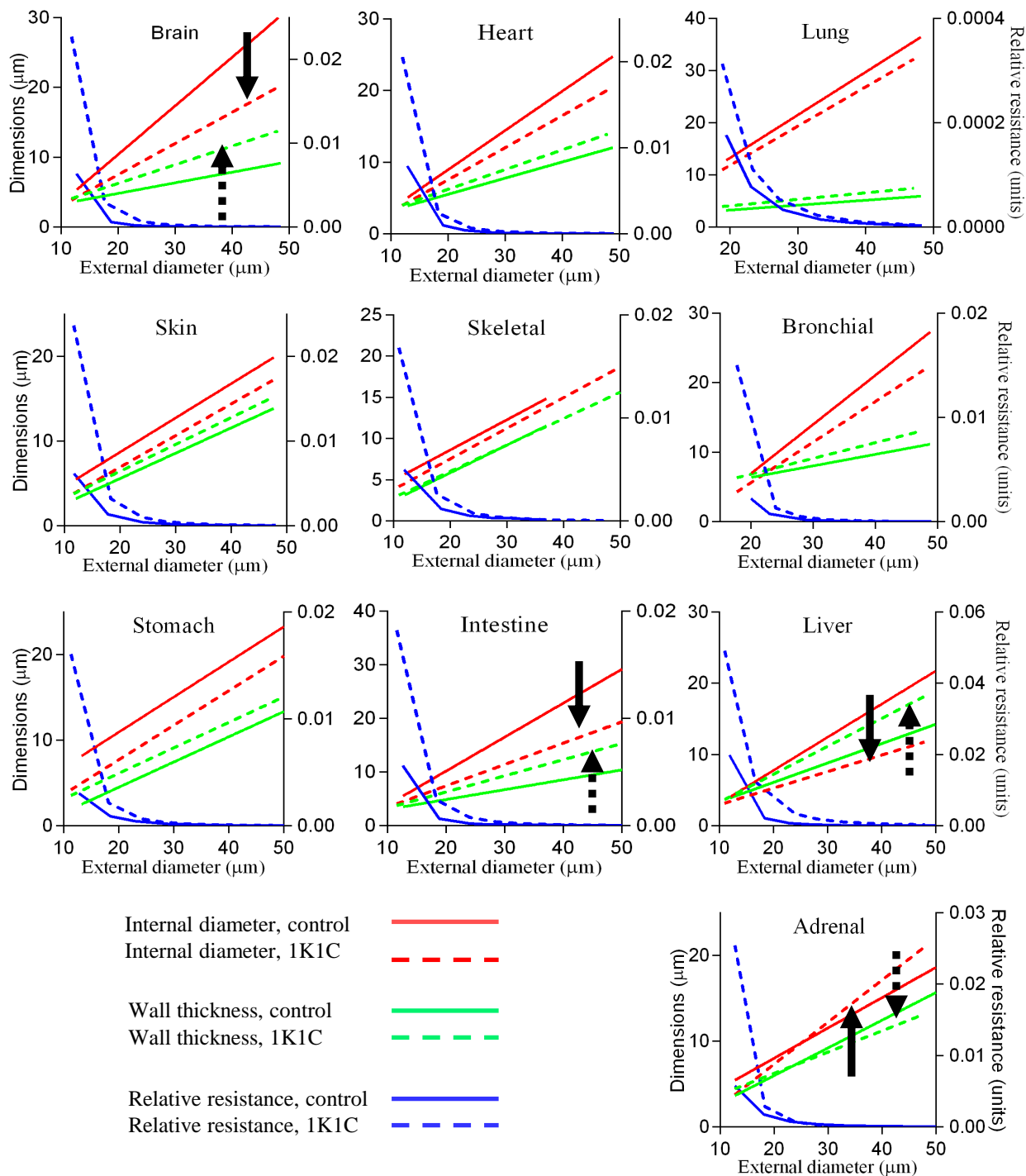

**S6 Fig.**

Supplement: S6 Fig — The internal diameter slopes decreased (solid arrows), and the wall thickness slopes increased (dashed arrows). Adrenal arteries demonstrated opposite directions. Complementary graphs to Fig 12B. (PDF) [file pone.0216734.s011.pdf]

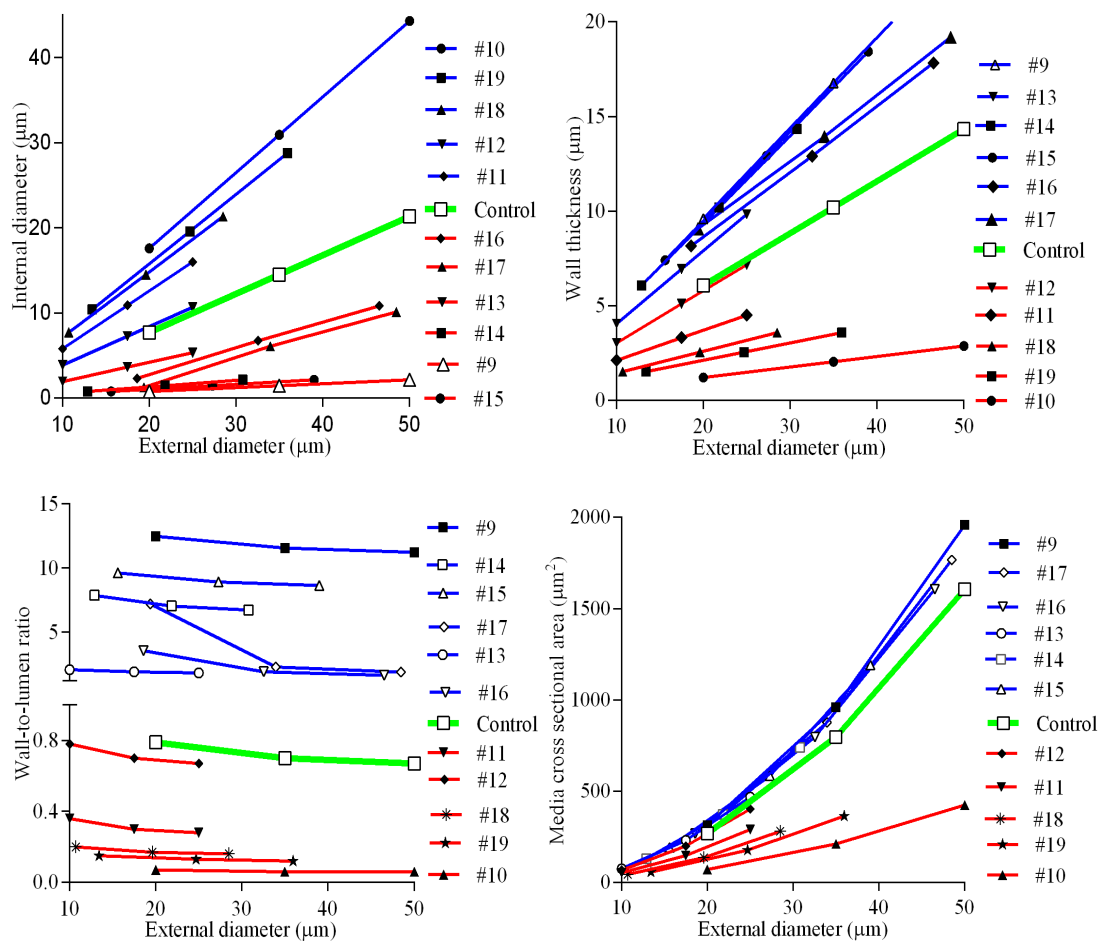

**S7 Fig.**

Supplement: S7 Fig — Displacement of linear regression lines up or down for any parameter was similar for many remodeling variants. Complementary graphs to Fig 13. (PDF) [file pone.0216734.s012.pdf]

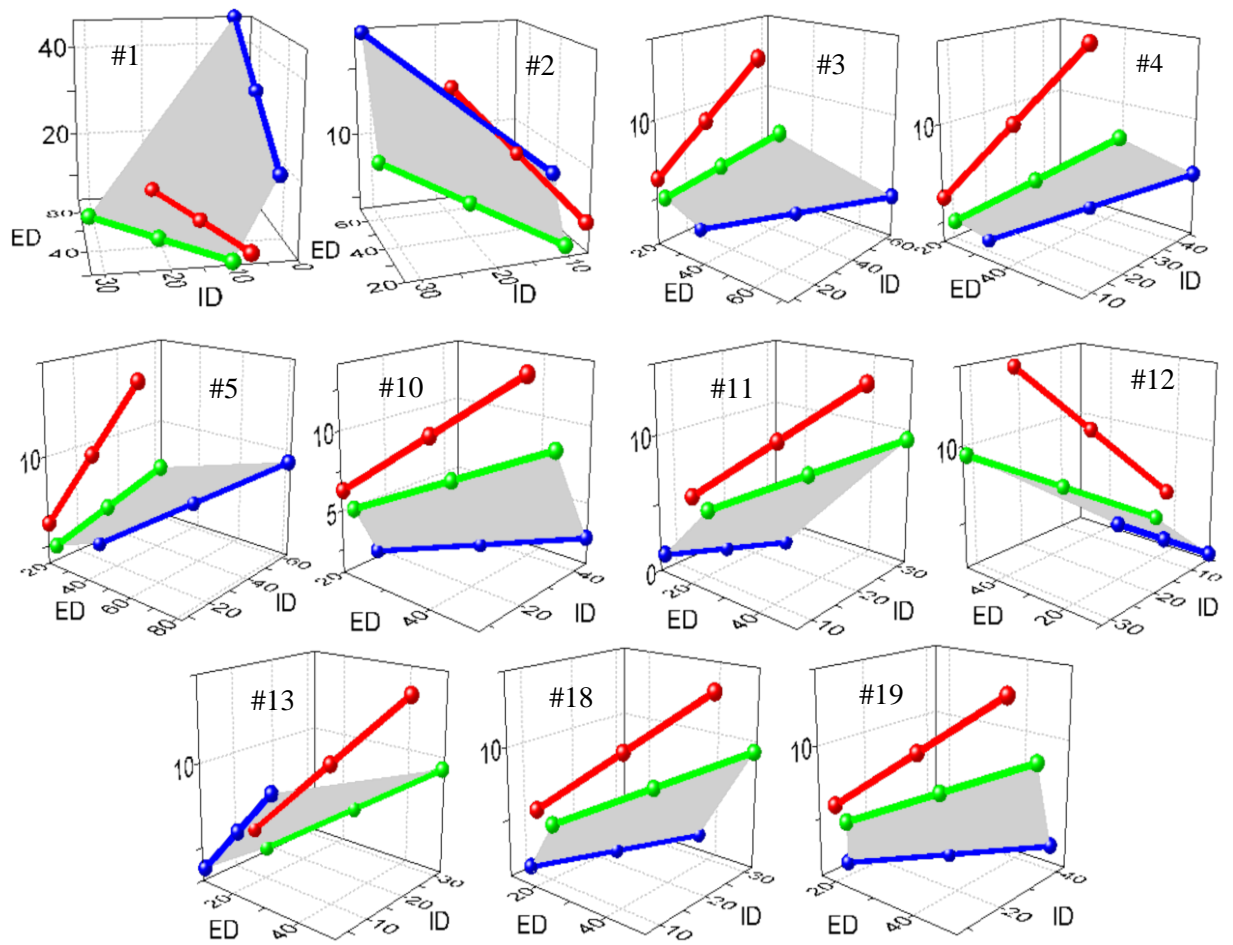

**S8 Fig.**

ED – external diameter; ID – internal diameter; WTh – wall thickness.

Supplement: S8 Fig — No variants were congruent. Complementary graphs to Fig 14. (PDF) [file pone.0216734.s013.pdf]
